# Engineered Tissue Models to Decode Host–Microbiota Interactions

**DOI:** 10.1002/advs.202417687

**Published:** 2025-05-14

**Authors:** Miryam Adelfio, Grace E. Callen, Xuesong He, Bruce J. Paster, Hatice Hasturk, Chiara E. Ghezzi

**Affiliations:** ^1^ Department of Biomedical Engineering University of Massachusetts‐Lowell Lowell MA 01854 USA; ^2^ ADA Forsyth Institute 245 First St Cambridge MA 02142 USA

**Keywords:** dysbiosis, female reproductive tract, host‐microbiome interactions, intestine, oral, skin, tissue models

## Abstract

A mutualistic co‐evolution exists between the host and its associated microbiota in the human body. Bacteria establish ecological niches in various tissues of the body, locally influencing their physiology and functions, but also contributing to the well‐being of the whole organism through systemic communication with other distant niches (axis). Emerging evidence indicates that when the composition of the microbiota inhabiting the niche changes toward a pathogenic state (dysbiosis) and interactions with the host become unbalanced, diseases may present. In addition, imbalances within a single niche can cause dysbiosis in distant organs. Current research efforts are focused on elucidating the mechanisms leading to dysbiosis, with the goal of restoring tissue homeostasis. In vitro models can provide critical experimental platforms to address this need, by reproducing the niche cyto‐architecture and physiology with high fidelity. This review surveys current in in vitro host–microbiota research strategies and provides a roadmap that can guide the field in further developing physiologically relevant in vitro models of ecological niches, thus enabling investigation of the role of the microbiota in human health and diseases. Lastly, given the Food and Drug Administration Modernization Act 2.0, this review highlights emerging in vitro strategies to support the development and validation of new therapies on the market.

## Graphical abstract

Host–Microbiota interactions in the human body. *Created in BioRender. Ghezzi, C. (2025) https://BioRender.com/ihivskg*.

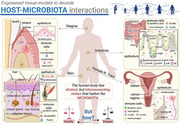

## 1 Introduction

The human microbiota, which includes bacteria, bacterial viruses, archaea, and fungi, is housed in distinct niches of the human body (e.g., skin, oral cavity, intestine, lungs, female reproductive tract (FRT)), with the gut harboring the highest abundance of microbial communities and the female reproductive tract as the lowest.^[^ ^1^ ^]^ Within each niche, the microbiota is unique or with minimal overlap, and represents a fingerprint of an individual, albeit the overall composition is quite similar in humans given their co‐evolutionary traits.^[^ ^2^ ^]^ Studies of the distribution and functions of the microbiota across niches have resulted in increased evidence of microbial communities’ key role in the human body's homeostasis.^[^ ^1^, ^2^ ^]^ Interactions among these microbial communities have been shown to shape host tissue physiology, participate in organ development, contribute to mucosal barrier integrity, body metabolism, immune regulation, and activation, and generally affect human health.^[^ ^1^, ^2^, ^3^ ^]^ While these interactions benefit the host and its niche‐specific microbiota, a systemic communication between microbial communities throughout the human body also exists, indicating long‐distance effects beyond individual ecological sites.^[^ ^1a^ ^]^ Local microbial niches can influence distant organs of the human body (e.g., intestine–brain, oral–intestine, or oral–intestine–brain, intestine‐FRT, or intestine‐skin) through various pathways, including metabolites, hormones, circulatory or immune systems. Among the major players involved in the homeostatic balance between the host and its microbial community, native immunity plays an important role in contributing to the stability of the ecosystem under healthy conditions.^[^ ^4^ ^]^ However, when a microbiota becomes dysbiotic, no longer in homeostatic equilibrium with the host, a shift in the relative abundance of species can occur, triggering competition within the community between commensals and pathogens, eventually resulting in the disruption of the local tissue barrier.^[^ ^2d^ ^]^ Recent research findings have linked pathological conditions of several organ systems to dysbiotic microbiomes and unbalanced interactions with the host, including periodontal disease, inflammatory bowel disease (IBD), eczema, or bacterial vaginosis.^[^ ^1^, ^2^, ^3^ ^]^ Although dysbiosis may remain localized at the tissue interface, evidence suggests that when dysbiotic communities thrive, systemic infection or inflammation might follow. Dysbiotic microbial communities of individual sites can act as reservoirs for opportunistic pathogens and contribute to a wide range of diseases by causing dysbiosis and impairing tissue function in distal niches through pathogen invasion or release of metabolites and signaling molecules into the bloodstream (e.g., Alzheimer's disease, blood‐brain permeability, microglia alterations, or arthritis).^[^ ^1^, ^2^, ^5^ ^]^ Despite the correlation of disease states with dysbiosis of the associated microbiota strongly supports the need to dissect the interplay between the human tissues and microbiota, there is still no clear understanding of the mechanisms of onset or progression of these conditions, nor of the exacerbation of pathology at distal sites. Understanding the ways in which these communities cooperate and by what means these interactions affect host physiology will provide valuable insight into how the collective microbiota shapes health and disease and contributes to the prevention and treatment of a wide range of human conditions.

To elucidate the mechanisms that regulate microbe‐microbe and host–microbiota interactions under eubiotic (healthy) and dysbiotic (disease) states, and in the transition between health and disease, in vitro tissue platforms have been recently explored as suitable approaches. These models can recapitulate the biological complexity of the phenomena governing host–microbiota interactions in controlled and simplified manners, yet preserving the human clinical relevance of the tissue interface of interest (e.g., oral, skin, intestinal, or FRT).^[^ ^6^ ^]^ These in vitro strategies represent a novel approach to clinical translational research, as they aim to address unmet needs and bridge the gap between bench side and clinical science. Considering the technological agility and cost efficiency of high‐throughput tools associated with in vitro models, these platforms would be critical in testing new hypotheses, diagnostic tools, intervention strategies for targeted diseases, or preventive medicine to preserve health conditions, accelerating animal research and clinical trials. In this regard, the Food and Drug Administration (FDA) Modernization Act 2.0,^[^ ^7^ ^]^ passed on December 29, 2022, authorized the use of cell‐based assays and computer models to investigate drug safety and effectiveness in an effort to eliminate the need for animal studies in preparation for human clinical trials.^[^ ^8^ ^]^ This aims to streamline drug development processes, potentially lowering costs and accelerating the availability of new therapies on the market. The introduction of these bench‐top platforms represents a paradigm shift in regulatory science, aligning with broader efforts to improve efficiency, ethical standards, and scientific rigor in pharmaceutical research and development. Specifically, in vitro modeling can provide important tools to significantly boost research progress in the context of host–microbiota interactions, by favoring precise control over experimental conditions.^[^ ^9^ ^]^ Yet, there are still refinements to be made to achieve the level of biological complexity displayed in vivo, such as the integration of full‐thickness tissue architecture, immune cells, and sustained long‐term culture systems in the presence of commensal/pathogenic bacteria.

This review aims to provide a comprehensive overview of current in vitro strategies to study host–microbiota interactions in some of the major tissue niches of the human body (skin, gingiva, intestine, and FRT (vaginal). Emphasis will be placed on describing the key characteristics of the host and microbiota in each tissue compartment and how research advances have aligned with clinical studies (human patients) in effort to elucidate disease onset/progression and accelerate the availability of new therapies on the market. This review will also discuss future perspectives emphasizing the importance of developing in vitro strategies stemming from multidisciplinary efforts to elucidate host–microbiota interactions during tissue homeostasis as well as homeostatic imbalances; a conclusive outlook will cover the development of multi‐organ models to study communication between niches and their role in human body functions, but also in disease onset and progression.

## 2 Considerations on In Vitro Tissue Modeling Design to Underscore Host–Microbiota Interactions

Past studies have shown that successful in vitro host–microbiota interaction models require a design that can recapitulate the cyto‐architecture, mechanical forces, and physiological properties and functions of the tissue (e.g., barrier formation, immune and pro/anti‐inflammatory responses, antimicrobial activities (e.g., peptides, mucus) or gradients (e.g., pH, oxygen, metabolites)).^[^ ^10^ ^]^ Host cyto‐anatomical complexity is achieved by employing different cell types (e.g., epithelial, stroma, endothelium, resident and circulating immune and nerve cells) obtained from stem cell differentiation protocols, commercially available primary cells, or isolated from patients’ biopsies or, immortalized cell lines. Immortalized cell lines have proven useful in in vitro and translational studies because of their longer life span; however, in some tissues, immortalized cell lines have failed to replicate some functional tissue responses, consequently limiting clinical translation. For example in the case of immortalized gingival fibroblasts (hTERTs), the challenge with *P. gingivalis* fails to trigger the upregulation of inflammatory mediators.^[^ ^11^ ^]^ Host cells are prepared using conventional culture techniques (e.g., culture flasks, well plates, or tissue inserts) or tissue engineering strategies (e.g., 3D scaffolds, bioprinting, organotypic cultures or organoids), each presenting opportunities and challenges, as reviewed in detail in.^[^ ^12^ ^]^ The microbial components of the in vitro model, instead, can be isolated from different tissue compartments, such as skin, saliva, dental plaque, feces, or FTR, and introduced in proximity to the epithelial tissue. Alternatively, synthetic biofilms prepared from single or multiple known microbial species can be used.^[^ ^13^ ^]^ The individual bacterial species used for biofilm formation can be cultured individually in test tubes, flasks, or Petri dishes and then combined before inoculation; however, the limitation of this approach lies in the lack of spatial and functional properties of the endogenous microbial community.^[^ ^10^, ^14^ ^]^

In the human body, the epithelium‐microbiota interface at different niches varies in epithelial morphology (e.g., squamous, columnar, or cuboidal), organization (e.g., single, pseudostratified, or stratified) and polarity,^[^ ^15^ ^]^ as well as microbial composition.^[^ ^3^, ^16^ ^]^ The microbiota is shaped by spatial profiles, governed by biogeographical parameters of the tissue niche (e.g., oxygen, pH, metabolite availability, adhesion proteins, mucus type, and thickness), which drive adhesion, organization, and biofilm composition.^[^ ^3^, ^16^ ^]^ Although current organotypic host–microbiota in vitro models replicate physiological interactions, they fail to maintain tissue functionality over long‐term culture period (>24h), due to bacterial growth rate, nutrient depletion, and cytotoxicity caused by waste accumulation.^[^ ^17^ ^]^ Moreover, given the varying oxygen tolerance of the organisms that comprise the microbiota, culturing complex biofilms in fully oxygenated environments reduces bacterial diversity over time, misrepresenting the in vivo interactions within the niche.^[^ ^2^, ^3^, ^10^ ^]^ Given the complexity of re‐creating each ecological niche, several considerations must be factored into the choice of the biomaterial and associated biofabrication technologies replicating host tissue features with high fidelity.^[^ ^18^ ^]^ Commercially available two‐dimensional or three‐dimensional culture systems employ tissue inserts (Transwell®) composed of a porous membrane (typically 0.4 µm), in which the epithelium‐microbiota interface is replicated by micro‐pipetting the microbiota onto the epithelium compartment. Such technologies have also been used in organoid cultures with reverse polarity (apical‐out), avoiding the time‐consuming and low‐throughput microinjection of microbiota into the organoid body (apical side).^[^ ^15^ ^]^ Replication of native extracellular matrix (ECM) structural and functional organization in these platforms is achieved via coating with collagen, poly‐L‐lysine, fibrin ECM (2D platforms), or 3D‐based hydrogel matrices (collagen, Matrigel).^[^ ^10^, ^19^ ^]^ Further advances in host morphological and functional features have been accomplished through the fabrication of functionalized hydrogels using self‐assembled peptides that do not require cross‐linking agents and mimic the nano‐macro‐ECM architectures to support cell responses.^[^ ^20^ ^]^ These hydrogels can also be fabricated by blending multi‐natural ECM proteins (e.g., collagen, fibrin, fibronectin) to model stiffness gradients to support the natural layering and polarity of host cells through their modular design.^[^ ^21^ ^]^ Endogenous collagen production can be achieved through non‐traditional tissue engineering strategies by the use of Whatman paper; once deposited, these collagen sheets can be further supplemented with microspheres for improved mimicking of native ECM composition and structural architecture.^[^ ^22^ ^]^ Overall, these biofabrication strategies, with their precise control of the cellular microenvironment through the formation of distinct epithelial structures and the integration of specific extracellular matrix components and signaling molecules, have great potential for studying microbiota colonization and interactions with host mucosal surfaces.^[^ ^23^ ^]^ Improvement of complex tissue architecture can be achieved through scaffolding techniques via natural (e.g., collagen, elastin, fibrin, hyaluronic acid, or silk fibroin) or synthetic polymers ((poly‐L‐lactic acid and poly (L‐lactide‐co‐caprolactone or polyglycolic acid (PLA/PLGA), which allow the tunability of mechanical properties, controlled degradation rates, and incorporation of multiple cellular populations.^[^ ^24^ ^]^ Dynamic systems, such as organ‐on‐a‐chip or bioreactors, coupled with mucosa‐equivalent interfaces (coatings, hydrogels, or scaffolds) are currently being optimized to incorporate native tissue forces (e.g., shear, peristalsis, traction, stiffness, pressure)^[^ ^10^, ^25^ ^]^ to provide biological significance, mass transport, metabolic waste elimination for long‐term investigations. Mechanical stimulation has been shown to enhance cellular proliferation, motility, or epithelium stratification and barrier, allowing the development of a more robust and physiologically relevant tissue model.^[^ ^10^, ^25^ ^]^ Furthermore, the integration of mechanical stimuli and physical conditions favors the maintenance of a controlled‐oxygenated micro‐environment, replenishment of nutrients, elimination of waste products, and control of bacterial growth.^[^ ^10b,h,i^ ^]^ Such organ‐on‐a‐chips have been largely developed for intestine models^[^ ^10b^ ^]^ via regulation of oxygen gradients for anaerobic species, but also in the oral niche.^[^ ^10^, ^26^ ^]^ However, the design of these culture platforms requires optimization for tissues to be cultured at the air‐liquid interfaces (e.g., gingiva, lung, or skin), as the epithelium‐microbiota interface is constantly submerged in the culture media, causing misrepresentation of the in vivo features of the niche. Emerging long‐term approaches are currently being optimized by using tissue engineering strategies, to model the anatomical architecture of the tissue, in conjunction with bioreactors designed to house and grow the tissue at air‐liquid interfaces while preserving the complexity of the microbiota.^[^ ^25c,d^ ^]^

The following sections will describe four relevant tissue compartments of the human body (in sequential order: skin, gingiva, intestine, and FRT (vagina)). The skin constitutes the first line of defense in the human body, as it offers a physical and chemical barrier that protects the host from the environment (chemicals, pollutants) and from colonization and invasion of external pathogens.^[^ ^27^ ^]^ Several connections between the oral and intestinal niches have been supported;^[^ ^1^ ^]^ failure of the periodontal sulcus surveillance system combined with microbiota action has been associated with intestinal dysbiosis and neurodegenerative diseases.^[^ ^1^, ^28^ ^]^ Most recently, awareness has grown for the FRT, especially in the context of sexually transmitted diseases.^[^ ^3b^ ^]^ Each niche will be described according to the following outline: 1) description of the cyto‐anatomical architecture of the tissue, with emphasis on the structural, physical, physiological, and mechanical features of the niche harboring the microbiota (biomass, composition, metabolism, oxygen, or pH and mechanical forces); 2) current state‐of‐the‐art of the mechanistic understanding of the host‐microbial interaction in healthy (eubiosis) and diseased (dysbiosis) states; 3) recent in vitro strategies and associated readouts aimed at replicating site‐specific host–microbiota interactions; 4) emerging clinical strategies targeting host–microbiota interactions in disease states.

## 3 Host–Microbiota Interactions: Microbial Niches

### 3.1 Skin

#### 3.1.1 Cyto‐Anatomical Architecture of the Skin: Host

**Figure 1:**
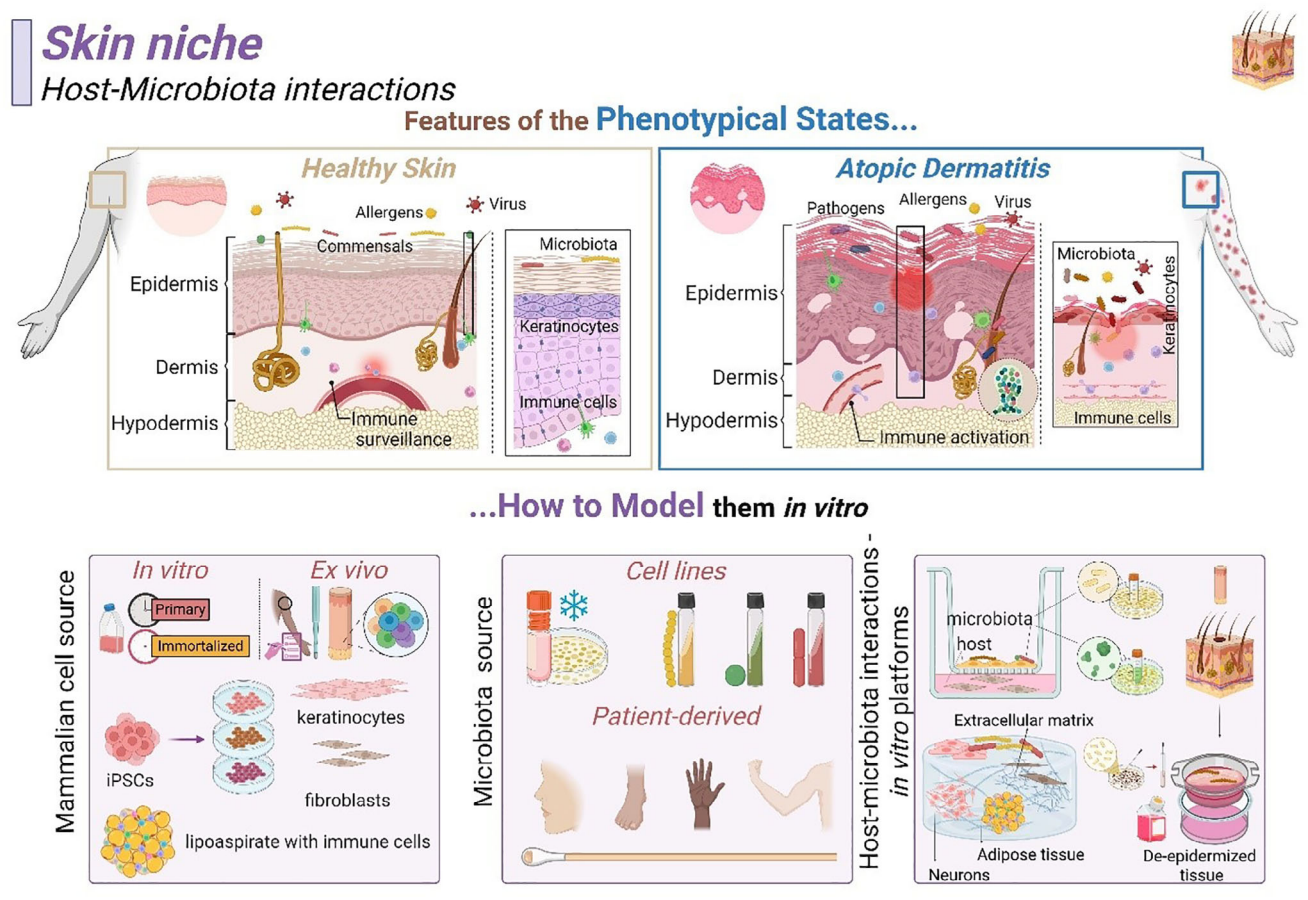
Skin niche. Top – Representation of the skin niche in healthy and disease (Atopic Dermatitis) states. Bottom – Overview of the mammalian and microbiota sources employed in current in vitro strategies. Created in BioRender. Ghezzi, C. (2025) https://BioRender.com/tnkynj1.

The skin (**Figure** **1**) is the largest barrier in the human body^[^ ^29^ ^]^ and is composed of the epidermis, dermis, and hypodermis (**Table** **1**).^[^ ^27^, ^30^ ^]^ The epidermis is the outermost layer of the skin and comprises five sublayers (basale, spinosum, granulosum, lucidum, and corneum), forming a physical barrier that protects the host from foreign pathogens, external environment, lifestyle factors, and dehydration.^[^ ^27^, ^30^ ^]^ The cell population residing in the epidermis is characterized by melanocytes, Merkel and Langerhans cells (layer basale), corneocytes (layer corneum), and keratinocytes (layer basale to lucidum), the latter being the most abundant and responsible for keratin production and lipid storage for tissue hydration.^[^ ^30^, ^31^ ^]^ The epidermis is connected to the dermis through a basement membrane rich in extracellular matrix proteins (collagens, laminin, glycoproteins, and proteoglycans).^[^ ^30a^ ^]^ The dermis, composed of two layers (papillary and reticular), is highly vascularized and innervated, and houses sweat glands and pilosebaceous unit (hair shaft, hair follicle, and sebaceous glands).^[^ ^30^ ^]^ Connective tissue is the main constituent of the dermis, consisting of collagen, elastin, fibronectin, and hyaluronic acid. It is populated by dermal fibroblasts and immune cells, including neutrophils, macrophages, dendritic cells, mast cells, eosinophils, B‐ and T‐lymphocytes (**Table** **2**). In addition, due to its greater degree of vascularization, the dermis plays a role in thermoregulation, oxygen supply to the epidermis, and waste elimination.^[^ ^30^ ^]^ The hypodermis is the last layer of the skin that anchors the dermis to the muscles and bones.^[^ ^30a^ ^]^ The hypodermis harbors primarily adipose tissue alongside large blood vessels and nerves, and plays a role in glucose regulation, angiogenesis, and inflammation.^[^ ^32^ ^]^

**Table 1:** Host tissue biological functions and assessments.

Biological functions and quantification metrics		Ecological niche				
		Skin	Oral	Intestine	FRT	Refs.
Biological Functions		Epidermis: Barrier, Immune response, Lipid Storage, Keratin production.	JE, SE: Attachment (to tooth), Immune response.	Mucosa/Submucosa: Absorption, Barrier, Hormone Secretion, Immune response, Secretion, Innervation Lymphatic drainage, Nutrient supply, Structural support, Vasculature.	Epithelium: Barrier function, Maintenance of pH, Regeneration, Immune response.	^[^ ^1^, ^3^ ^,^ ^3^, ^4^ ^,^ ^10^, ^27^, ^30^, ^31^, ^32^, ^71^, ^72^, ^73^, ^74^, ^75^, ^76^, ^77^ ^,^ ^81^, ^106^, ^107^, ^108^, ^109^, ^110^ ^,^ ^151^, ^152^, ^153^, ^154^, ^155^, ^156^, ^157^, ^219^ ^]^
		Dermis: O_2_ transport, Thermoregulation, Waste elimination	OGE: Immune response, Protective barrier.	Muscularis propria: Peristalsis, Segmentation, Contractions, Sphincter function, Immune response.	Lamina Propria: Immune response, Nutrient supply, Structural support.	
		Hypodermis: angiogenesis, glucose regulation, inflammation.	Lamina propria: Immune response, Nutrient supply, Structural support.	Serosa: Lubrication, Structural support, Immune response.	Muscularis/Adventitia: Contraction/Relaxation, Structural support, Vasculature, Innervation.	
*Assessments*	*Readouts*					
*Barrier Integrity*	TEER					
	Gene expression	keratins: 5,14^[^ ^219a^ ^]^	mucins^[^ ^73^ ^]^	occludin^[^ ^219g^ ^]^	MMP, mucins^[^ ^219n^ ^]^	
	Lipid analysis					
	LDH release					
*Tissue Layer Organization and Differentiation*	Diffusion/Permeation assays					
	Histology	H&E	H&E	H&E	H&E	
	Immunohistochemistry					
	Microscopy	SEM, CLSM	SEM, CLSM	SEM, CLSM	SEM, CLSM	
	Gene expression	keratins: 1,10^[^ ^219a^ ^]^	cadherins^[^ ^73^ ^]^	mucins^[^ ^219m^ ^]^	involucrin^[^ ^10a^ ^]^	
*Mechanics and Elasticity*	Tensile, compressive, or shear tests					
*Inflammatory Response*	Cytokine quantification ‐ *Pro/anti‐inflammatory*	IL‐1α, IL‐1β, IL‐4^[^ ^219q^ ^]^	IL‐6, IL‐10, IL‐37, TNF‐α^[^ ^219k^ ^]^	IL‐10, TNF‐α, INF‐γ^[^ ^219^, ^220^ ^]^	IL‐1RA, IL‐1β, IL‐6^[^ ^219c^ ^]^	

Within each niche are examples of biological indicators and techniques. Pastel colors represent the respective niche and the type of readouts applicable. Gray sections indicate that the proposed reading cannot be applied to the respective niche. Abbreviations: TEER, transepithelial electrical resistance; LDH, lactate dehydrogenase; H&E, Hematoxylin and Eosin; SEM, scanning electron microscopy; CLSM, confocal laser scanning microscopy; MMPs, matrix metalloproteinases.

**Table 2:** Spatial localization of immune cells in host tissue and their biological functions.

Tissue localization and cellular population	Biological functions	Refs.
*Skin*		
B‐, T‐cells Basophil Dendritic cell Eosinophils Langerhans Macrophage Mast cells Neutrophil NK cell Plasma cell^[^ ^37^ ^]^	Epidermis ‐ Physical barrier to prevent pathogen invasion ‐ Initiates innate immune responses ‐ Regulation of antimicrobial peptide secretion	[27, 30, 31, 32, 221]
	Dermis ‐ Physical barrier support ‐ Responsible for innate and adaptive immune response and regulation ‐ Orchestrates cytokine network regulation ‐ Antigen presentation to the immune system	
*Oral*		
B‐ and T‐cells Dendritic cell Langerhans Intraepithelial lymphocytes Macrophages Neutrophils^[^ ^38^ ^]^	Non‐keratinized epithelium (JE, SE) ‐ Physical barrier to prevent pathogen invasion ‐ Initiates innate immune responses ‐ Regulation of antimicrobial peptide secretion	[71, 72, 73, 74, 75, 76, 77, 222]
	Keratinized epithelium (OGE) ‐ Physical barrier support ‐ Responsible for innate and adaptive immune response and regulation	
	Lamina propria ‐ Physical barrier support ‐ Immune cell composition and cytokine production	
*Intestine*		
Dendritic cells Eosinophils Lymphocytes Macrophages Mast cells Paneth cells Plasma cells T‐cells^[^ ^38^ ^]^	Mucosa ‐ Physical barrier to prevent pathogen invasion ‐ Initiates innate immune responses ‐ Regulation of antimicrobial peptide secretion	[1, 3, 4, 110, 223]
	Submucosa ‐ Immune cell composition and cytokine production ‐ Protect from pathogen invasion while allowing food antigens and commensal bacteria enter	
	Muscularis Propria ‐ Phagocytose and clear pathogens, antigens to maintain tissue homeostasis	
	Serosa ‐ Maintains local homeostasis and tissue damage repair	
*Female Reproductive Tract*		
B‐ and T‐cells Dendritic cells Langerhans cells Macrophages Mast cells Monocyte Natural killer cells Neutrophils^[^ ^39^ ^]^	Epithelium ‐ Physical barrier to prevent pathogen invasion. ‐ Initiates innate immune responses. ‐ Regulation of antimicrobial peptide secretion	[3, 151, 152, 153, 154, 155, 156, 157, 224]
	Lamina Propria ‐ Physical barrier support ‐ Immune cell composition and cytokine production	

Abbreviations: NK cell, natural killer cell; JE, junctional epithelium; SE, sulcular epithelium; OGE, oral gingival epithelium.

#### 3.1.2 Cyto‐Anatomical Architecture of the Skin: Microbiota

The epidermis and dermis house the skin microbiota with a biomass (microbes/person) of approximately 10^11^ (Figure 1).^[^ ^6^, ^27^, ^33^ ^]^ Predominantly, the skin comprises four bacterial phyla (*Actinobacteria, Firmicutes, Proteobacteria*, and *Bacteroides*), together with fungi and viruses.^[^ ^27^, ^34^ ^]^ Analysis of bacterial diversity on the skin microbiota has highlighted interpersonal variation across the human population, but also across skin sites (e.g., sebaceous face, moist elbow, or dry palm),^[^ ^27^, ^34^ ^]^ suggesting a high degree of adaptation and coevolutionary mutualism between host and microbial communities.^[^ ^27^ ^]^ Across skin regions, complex physical and chemical properties, such as temperature, pH, sebum content or moisture and oxygen, in conjunction with the cyto‐anatomical characteristics of the epidermis and dermis, individual habits, and environmental exposure, drive the assemblage and composition of different microbial ecosystems on the skin sites.^[^ ^27^, ^34^, ^35^ ^]^ Examples are *Propionibacteria* and *Staphylococcus* species, abundant in sebaceous sites, or *Corynebacterium* species, predominant in moist sites.^[^ ^27^, ^34^ ^]^

In comparison to other niches in the human body, the skin microbiota presents a low biomass and is characterized by resident taxa (e.g., *C. acnes* or *S. epidermidis*), which stabilize in composition in specific skin sites of an individual only after puberty, due to fluctuations in sex hormones and sebum production by the sebaceous glands.^[^ ^3^, ^27^, ^34^ ^]^ Although most of the microbial population colonizes the skin surface (aerobic environment),^[^ ^36^ ^]^ the spatial architecture of the pilosebaceous unit and sweat glands creates microaerophilic regions with anoxic to hypoxic microenvironment that allow anaerobic or facultative commensal species to thrive with limited interspecies competition.^[^ ^3^, ^27^, ^36^, ^37^ ^]^ In addition to providing a hypoxic environment, the pilosebaceous unit is also fundamental in influencing the structural ecology of the skin niche; it has in fact enabled the evolutionary selection of bacterial species metabolizing lipid‐rich sebum, salts, or cellular debris,^[^ ^3^, ^27^ ^]^ whose role is important for maintaining acidic pH in healthy skin, keratinocytes proliferation, barrier integrity, and modulation of immune response during homeostasis or wound healing.^[^ ^3^, ^27^ ^]^

#### 3.1.3 Skin Host–Microbiota Interactions: Eubiosis and Dysbiosis

The balanced skin microbiota (eubiosis) is fundamental in preserving the healthy state of human skin.^[^ ^3^, ^27^ ^]^ Although human skin is a dry, slightly acidic environment with low nutrient availability,^[^ ^27^, ^35^ ^]^ commensals have developed strategies to survive and persist while interacting with hosts and contributing to host physiology.^[^ ^27b^ ^]^ Eubiotic skin microbiota strengthens the skin barrier by promoting the differentiation of keratinocytes and preserving the barrier structural integrity, acting against the colonization and invasion of pathogens.^[^ ^27^, ^38^ ^]^ Moreover, the skin microbiota is pivotal in stimulating the skin innate and adaptive immune responses by eliciting the host's production of antimicrobial peptides or T‐cells activation against foreign pathogens.^[^ ^27^, ^38^ ^]^ For instance, the metabolism of sebum into free fatty acids, or corneocyte debris by bacteria inhabiting the pilosebaceous unit (e.g., *Cutibacterium acnes* (reclassification of *Propionibacterium acnes*) and other *Corynebacterium* spp.) contributes to skin's resistance to pathogen colonization. This is achieved by augmenting the skin's chemical barrier and eliciting immune defense through neutrophil recruitment and release of host‐derived antimicrobial peptides (e.g., human cathelicidin LL37 or β‐defensins).^[^ ^3^, ^27^, ^38^ ^]^ Along with host–microbiota interactions, the preservation of healthy skin is supported by microbial communities’ communication. Quorum sensing or protease production are examples of diverse mechanisms adopted by the microbial ecosystem to inhibit the growth of adverse pathogens.^[^ ^3a^ ^]^ Therefore, host–microbiota and microbe‐microbe interactions within the ecosystem are crucial in promoting skin health.

Well‐studied host–bacteria interactions are those with *C. acnes* or *Staphylococcus epidermidis*. *C. acnes* is an aerotolerant anerobic commensal that resides on the skin surface but also in the pilosebaceous unit; this bacteria is known to produce propionic acid, that contributes to the maintenance of the acidic pH of healthy skin, and to be involved in the innate immunity barrier via lipase secretion and the cascade associated with sebum metabolism.^[^ ^3^, ^27^ ^]^ *S. epidermidis* is a commensal facultative anaerobe that plays a central role in the communication between the immune system and the microbiota.^[^ ^27a^ ^]^ *S. epidermidis* promotes microbial colonization via the modulation of local inflammation and T‐cell function without eliciting inflammation, thereby educating the immune system on the presence of skin commensal bacteria and responding to alterations of the physiological skin microbiota, especially during the postnatal period.^[^ ^3^, ^27^, ^38^, ^39^ ^]^ Moreover, *S. epidermidis* modulates the host innate immune system during tissue repair and wound healing, preventing excessive skin damage due to inflammation and immune activation.^[^ ^3^, ^36^, ^38^ ^]^

Although there is a homeostatic relationship between host and microbiota in the skin, a decrease in nutrient availability or damage to the microbial ecosystem might cause alterations in host–microbiota interactions, leading to skin disorders (e.g., acne, chronic wounds, or atopic dermatitis) (**Table** **3**).^[^ ^3^, ^27^, ^29^ ^]^ Some skin dysbiotic conditions are linked to the increased abundance of single pathogens, such as *Staphylococcus aureus* in atopic dermatitis (AD), or commensals acting as opportunist pathogens, such as *C. acnes* in acne vulgaris (**Table** **4**).^[^ ^3^, ^27^, ^29^ ^]^ However, it is unclear whether dysbiosis is a disease trigger or a direct consequence.^[^ ^3^, ^27^ ^]^ AD is a noncontagious skin disease affecting 30% of the U.S. population^[^ ^40^ ^]^ and presents skin lesions accompanied by an intense itching sensation.^[^ ^41^ ^]^ The pathology is correlated with an increase in the relative abundance of the opportunistic pathogen *S. aureus*, primarily found in skin lesions,^[^ ^3^, ^27^, ^41^, ^42^ ^]^ and a decrease in the diversity of the skin microbiota (enrichment in *Streptococcus* spp. and *Gemella* spp., as well as depletion of *Dermacoccus* spp.).^[^ ^27a^ ^]^ Dysbiotic host–microbiota interactions during AD are associated with epidermal barrier impairment, skin pH variations, pro‐inflammatory responses, and temporal paralysis of some components of the immune system.^[^ ^3^, ^27^ ^]^ Specifically, *S. aureus* acts at different levels: 1) producing α‐toxin, that destroys the epidermal barrier by forming pores in keratinocyte cells; 2) stimulating keratinocytes to release proteases (kallikrein 6, 13, and 14) causing self‐impairment of the skin barrier; 3) favoring viral infection via α‐toxin production; 4) enhancing pro‐inflammatory mechanisms in keratinocytes through the TNF receptor, in monocytes (IL‐1β) and in CD4^+^T (IL‐17); 5) inducing T‐cell paralysis (block of proliferation and cytokine production), due to lipoteichoic acid exposure, a cell wall component of *S. aureus*.^[^ ^27^, ^38^, ^41^, ^43^ ^]^

**Table 3:** Common pathogens associated to H‐M diseases.

Niche	Pathogen	Disease	Mechanisms	Refs
Skin	*S. aureus*	Atopic Dermatitis	(1) aberrant epidermal lipid composition; (2) change of the pH (basic) of the skin; (3) cytolytic toxin production leading to initiation of pro‐inflammatory responses and temporary T‐cell paralysis	[225]
	*C. acnes*	Acne Vulgaris	(1) increased production of sebum; (2) epidermal barrier impairment; (3) abnormal keratinization	
Oral	*P. gingivalis*	Periodontal Disease	(1) adheres and invades epithelial cells by transient expression of gingipains; (2) induction of apoptosis; (3) impairment of intracellular persistence of host cells; (4) stimulates *T. denticola* growth	[226]
	*T. denticola*		(1) metabolites support *P. gingivalis* and accumulate in periodontal pockets; (2) epithelial barrier impairment; (3) regulation of inflammasome signaling	
	*T. forsythia*		(1) increases proteolytic activity (2) enhances abscesses formation and alveolar bone resorption	
Intestine	*E. coli*	Inflammatory Bowel Disease (CD and UD)	Adherence and invasion of epithelial cells, multiplying within macrophages	[227]
	*B. fragilis*		(1) Symbiont, production of sphingolipids to regulate homeostasis; (2) Promoting colonic mucosa proliferation and mucus secretion	[228]
FRT	*G. vaginalis*	Bacterial vaginosis	(1) Adheres to epithelial cells and aids the growth of anaerobes (2) Increase in pH	[229]
	*P. bivia*		(1) Increase in pH (2) Immune cell evasion – does not trigger inflammatory response in vaginal cells	[188a]

Abbreviations: H‐M, host–microbiota; S. aureus, Staphylococcus aureus; C. acnes, Cutibacterium acnes; P. gingivalis, Porphyromonas gingivalis; T. denticola, Treponema denticola; T. forsythia, Tannerella forsythia; E. coli, Escherichia coli; B. fragilis, Bacteroides fragilis; G. vaginalis, Gardnerella vaginalis; P. bivia, Prevotella bivia; FRT, female reproductive tract.

**Table 4:** Pathogen sociability involved in H‐M diseases.

Niche	Pathogens	Disease	Mechanisms	Refs.
Skin	*S. aureus – S. epidermidis*	Atopic Dermatitis	In non‐damaged epithelium *S. epidermidis* can *inhibit S. aureus* virulence, whereas in a damaged epithelium, *S. aureus* is able to overgrow	[225, 230]
	*C. acnes – S. epidermidis*	Acne Vulgaris	*S. epidermidis* enhances *C. acnes* anaerobic biofilm growth and formation	
Oral	*S. gordonii – P. gingivalis*	Periodontitis	(1) Regulation of consortia pathogenic potential; (2) regulation of gene encoding fimbrial adhesins; (3) increased gingipain activity and hemin acquisition	[81, 231]
	*P. gingivalis – T. denticola*		Mutualistic microbes, *P. gingivalis* stimulates *T. denticola* growth, and *T. denticola* metabolites support *P. gingivalis* growth	
	*T. forsythia – F. nucleatum*		Synergistically stimulate host responses and induce alveolar bone loss to a greater degree when together in comparison to when alone	[232]
Intestine	*E. coli – B. fragilis*	Inflammatory Bowel Disease	In concert, *E. coli* and *B. fragilis*, can exert a detrimental effect on the peritoneal host defenses of translymphatic absorption and bacterial phagocytosis.	[233]
FRT	*A. vaginae – P. bivia – G. vaginalis*	Bacterial vaginosis	Together, *A. vaginae* and *P. bivia* co‐incorporate without influencing overall biomass, while significantly effecting gene expression related to biofilm maintenance in G. vaginalis	[234]

Abbreviations: H‐M, host–microbiota; S. epidermidis, Staphylococcus epidermidis; S. gordonii, Streptococcus gordonii, F. nucleatum, Fusobacterium nucleatum; A. vaginae, Atopobium vaginae; FRT, female reproductive tract.

Even though it has been established that the skin benefits from mutualistic interactions with the microbiota, commensal bacteria can shift to pathogenic and cause skin diseases.^[^ ^3^, ^27^, ^38^, ^41^, ^43^ ^]^ Yet, many questions remain unaddressed regarding the biological mechanisms that drive dysbiosis and skin inflammation. Further investigations using human subjects or humanized in vitro models are needed to understand whether dysbiotic microbiomes are drivers of skin disorders or a consequence of inflammatory conditions, considering the limitations in the use of animal models with respect to human relevance.^[^ ^29^, ^44^ ^]^

#### 3.1.4 In Vitro Modeling of Skin Host–Microbiota Interactions

Several organotypic models (Figure 1) of healthy and diseased skin have been developed to facilitate microbial studies (**Table** **5**). Host models are mainly based on primary cultures of keratinocytes and fibroblasts obtained from skin biopsies and the outer sheath of hair follicles or based on immortalized cell lines.^[^ ^44^, ^45^ ^]^ Recapitulation of dermis and epidermis has been achieved by culturing keratinocytes as monocultures or co‐cultures with stromal cells embedded in collagen or fibrin matrices, or with de‐epidermized dermis of healthy skin.^[^ ^44^, ^46^ ^]^ As collagen matrices are susceptible to cell‐mediated contraction over time,^[^ ^47^ ^]^ alternative strategies favored natural or synthetic polymers, such as silk‐collagen blends or peptide‐based hydrogels, to support the long‐term culture of the epidermis and dermis in vitro.^[^ ^20^, ^48^ ^]^ While the design of peptide‐based hydrogel allowed the recapitulation of native skin extracellular matrix architecture via self‐assembly into helical nanofibers without the need of crosslinking agents, scaffolding techniques facilitated tissue robustness, enhancing in vitro tissue performances and favoring the incorporation of multiple cellular counterparts.^[^ ^20^, ^24^ ^]^ To contribute to physiological relevance and, thus, cellular complexity, further strategies have adopted induced pluripotent stem cells (iPSCs) to generate functional skin populations and shafts (e.g., keratinocytes, fibroblasts, endothelial cells, immune cells, and hair follicles) for skin tissue engineering using Matrigel or collagen gels.^[^ ^45^, ^49^ ^]^ Considering the cellular cross‐talk among skin layers for tissue maturation and metabolism, hypodermis, comprised of adipose tissue and the nervous system, has instead been incorporated into 3D in vitro model of natural polymers (silk fibroin, collagen) using patient‐donated abdominoplasty lipoaspirate and induced human neural stem cells (ihNSCs) obtained via reprogramming of dermal fibroblasts.^[^ ^48^, ^50^ ^]^ The above models successfully replicated the layered structure of the epidermis, epithelium proliferation (positive to Ki67 stratification) and differentiation (positive to Keratin or Loricrin), expression of junctional complexes, and responsiveness to pathogens. Skin cell viability and topographical features (multi‐layer organization, barrier function, permeability, or mass transport)^[^ ^51^ ^]^ have improved following flow stimulation in microfluid devices, which facilitates the incorporation of endothelial cells,^[^ ^52^ ^]^ immune cells,^[^ ^53^ ^]^ or hair follicles,^[^ ^54^ ^]^ thus enhancing the cyto‐complexity of skin models. The need for microfluidic devices stems from the need for improved skin cellular features as well as the incorporation of vascularization, which are essential to study skin angiogenesis or improved survival of skin grafts and, in general, to test drugs for drug–cell interactions in a more physiologic environment.^[^ ^52b^ ^]^ Most of these studies have been proven useful in investigating microbiota–skin interplay with patient‐derived microbiota^[^ ^10c,d^ ^]^ or selected bacterial cell lines.^[^ ^55^ ^]^ Commensal (e.g., *S. epidermidis* or *C. acnes*) or pathogenic (*S. aureus*) microbes were chosen to study colonization timeline, viability, and tissue response to microbial challenge.^[^ ^55a–c^ ^]^ Moreover, as immune cells are crucial in the preservation of healthy skin and response to pathogen colonization and invasion,^[^ ^27a^ ^]^ CD4^+^T‐cells, isolated from peripheral blood, have also been incorporated in the skin in vitro models (epidermis and dermis) to test the capacity of activated T‐cells to secrete cytokines in response to bacterial infections.^[^ ^56^ ^]^ Lastly, to support clinical translation, commercially available skin models have been developed to test skin irritants or corrosives (e.g., EpiSkin, EpiDerm).^[^ ^10^, ^30^, ^57^ ^]^ These models, together with commercially available skin wound models (e.g., Graftskin), are useful tools to study bacterial infection processes, such as adherence and invasion, and to identify pharmacological treatments to eradicate multidrug resistance.^[^ ^58^ ^]^ Collectively, functional host–microbiota readouts are centered on longitudinal assessments of epidermis architecture (i.e., stratification, differentiation, and barrier properties),^[^ ^51^, ^55^, ^56^ ^]^ microbial colonization,^[^ ^55^ ^]^ pro‐ and anti‐inflammatory responses,^[^ ^55^, ^56^ ^]^ and drug testing.^[^ ^51^, ^55^ ^]^

**Table 5:** Overview of host–microbiota organotypic features of in vitro models.

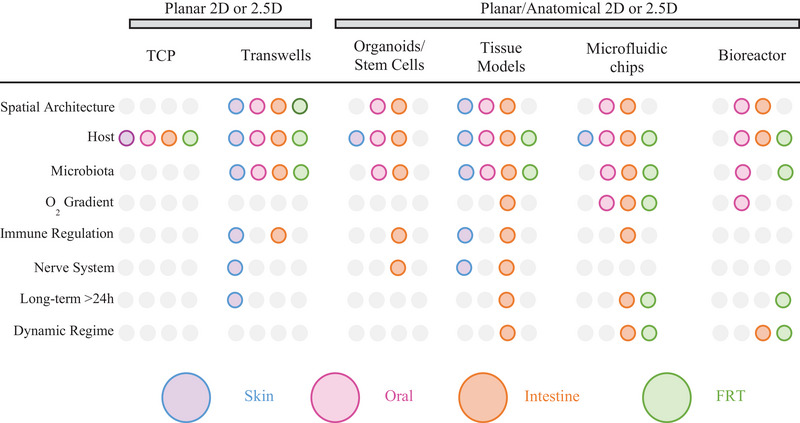

Abbreviations: TCP, tissue culture plate; H‐M, host–microbiota; FRT, female reproductive tract.

#### 3.1.5 Emerging Clinical Strategies Targeting Skin Host–Microbiota Interactions in Disease States

The FDA Modernization Act 2.0. pushes the boundaries of in vitro modeling to validate drug targets, assess efficacy and safety, explore drug interactions and resistance, and lastly support personalized medicine approaches, enhancing our understanding of disease pathogenesis.^[^ ^8^, ^38^, ^59^ ^]^ For tissue models to serve as validation platforms, physiological relevancy and phenotypical representations of skin disease states need to be represented. Current in vitro skin models lack the physiological complexity necessary for drug testing (e.g., vasculature, immunocompetence, innervation, secretory function, or microbiota ecosystem), posing concerns for the lifespan of the model and correlation with clinical conditions.^[^ ^27^, ^60^ ^]^ Research efforts targeting skin host–microbiota interactions are centered on two parallel strategies: 1) development of in vitro models of skin diseases as platforms for drug testing, and 2) utilization of the microbiota in disease treatment.^[^ ^27^, ^60^, ^61^ ^]^ Several disease models have been developed to test treatments for AD and Acne Vulgaris (AV), paving the way for the use of these platforms to elucidate disease mechanisms and treatment efficacy.^[^ ^61^, ^62^ ^]^ Examples of in vitro representation of AD traits, such as inflammatory mediators, skin barrier proteins, and gene expression biomarkers (e.g., involucrin, type IV collagen, and Ki67), were based on human iPSC‐derived skin organoids enriched with *S. aureus* or inflammatory dermal infiltrate, based on IL‐4 and IL‐13, in a de‐epidermized dermis model.^[^ ^61^, ^62^, ^63^ ^]^ Moreover, commercially available skin equivalents, such as EpiSkin, SkinEthic, and EpiDerm have demonstrated the ability to foster a complex microenvironment essential for the physiological modeling of skin microbiota‐related pathologies.^[^ ^10^, ^64^ ^]^ Available AD and AV clinical treatments include corticosteroids and antibiotics. Their prolonged usage has been shown to cause hormonal imbalance induced by systemic absorption, bacterial resistance to antibiotics, and a shift in the composition (diversity) of the skin microbiota, resulting in reduced drug efficacy over time and further exacerbating dysbiosis.^[^ ^65^ ^]^ To overcome these limitations, recent advancements are based on engineering commensal skin bacteria to deliver critical missing natural proteins (e.g., linolenic acid, chitosan)^[^ ^66^ ^]^ through the stratum corneum of the skin using solid lipid nanoparticles (SLNs) and nanostructured lipid carriers (NLCs).^[^ ^60^, ^67^ ^]^ This strategy, currently in clinical phase 1b and clinical phase 1/2, has been tested in in vitro diseased models recapitulating papulopustular rosacea, Netherton syndrome, chronic wounds, and diabetic foot ulcers.^[^ ^60^, ^67^ ^]^

Along with clinical representation of disease states, alternative methodologies focus on leveraging the microbiota to develop disease treatments. Precision medicine approaches are advantageous as they can target specific immune pathways or microbial imbalances, leading to more effective and tailored outcomes.^[^ ^38^, ^59^ ^]^ Examples of immune‐targeted biological therapies for AD currently available in the clinic and previously tested in vitro include dupilumab and tralokinumab, which, by acting on the IL‐4 and IL‐13 immune pathways, reduce the inflammatory state, improve the skin barrier, and support eubiosis of the skin microbiota.^[^ ^68^ ^]^ Microbiota‐target therapies are also based on bacteria manipulation via supplementation, augmentation, or suppression of microorganisms.^[^ ^29^, ^38^, ^69^ ^]^ These approaches allow more precise control over environmental factors contributing to dysbiosis and can be tested in vitro to develop tailored treatment regimens to each patient's specific microbiota composition to restore symbiosis, thereby reducing symptoms and increasing long‐term efficacy.^[^ ^38^ ^]^ Supplementation/augmentation/suppression strategies have been based on pre‐cultivation of low‐abundance microbes by optimization of culture conditions, as in the case of *C. acnes* grown in the hair follicles and involved in the pathogenesis of AV.^[^ ^10^, ^29^, ^37^, ^38^ ^]^ Other approaches include individualized supplementation of probiotics or consortia of microbiota, engineered live biotherapeutic products (eLBPs), and microbiota‐targeted dietary and lifestyle interventions, which directly influence an individual's unique microbiota or immune system.^[^ ^38^, ^59^, ^60^, ^69^ ^]^ By focusing on restoring natural microbial ecosystems rather than broadly targeting the related immune pathways, these modalities have the potential for longer‐lasting benefits by promoting healthy microbiota.^[^ ^29^, ^38^, ^59^, ^68^ ^]^

Beyond screening potential therapies and understanding microbiota interactions by studying disease mechanisms, in vitro modeling allows elucidation of differences in individual microbiome compositions to tailor treatments, based on genetic traits, environment, and lifestyle.^[^ ^59^, ^63^, ^68^ ^]^ This illustrates the potential of using in vitro modeling to streamline the clinical translation of therapeutics, as in the case of AD and AV, directly improving drug development efficiency.^[^ ^8^, ^27^, ^59^, ^65^, ^70^ ^]^

### 3.2 Gingival Tissue

#### 3.2.1 Cyto‐Anatomical Architecture of the Gingival Tissue: Host

**Figure 2:**
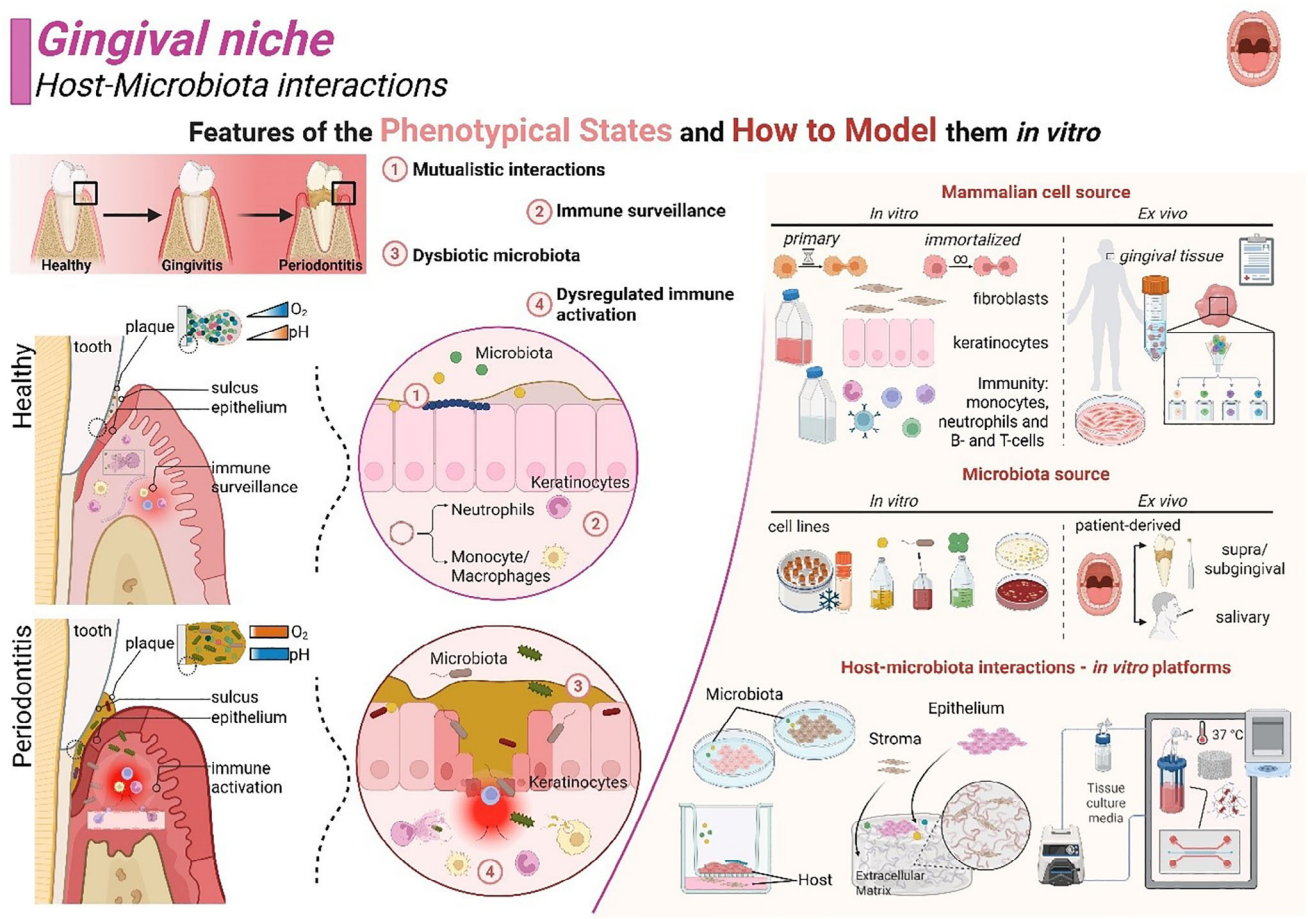
Gingival niche. Left – Representation of the gingival niche in healthy and disease (Periodontal Disease) states. Right – Overview of the mammalian and microbiota sources employed in current in vitro strategies. Created in BioRender. Ghezzi, C. (2025) https://BioRender.com/hwntv5x.

The human gingiva, along with the tissues comprising the oral mucosa (lining, masticatory, and specialized), (**Figure** **2**) represents the first line of defense against allergens and microorganisms before reaching systemically other tissues in the human body and potentially contributing to a wide range of diseases.^[^ ^71^ ^]^ As a part of the periodontium,^[^ ^72^ ^]^ the gingiva comprises epithelial and connective tissues lining the masticatory mucosa attached to the teeth and alveolar bone (Table 1).^[^ ^73^ ^]^ Given the continuous exposure to different mechanical stimuli and the proximity to the oral microbiota, the epithelial tissue presents a non‐keratinized junctional (JE) and sulcular (SE) epithelium, and a keratinized oral gingival epithelium (OGE), each presenting a different cyto‐anatomical structure (e.g., thickness, cytokeratin expression, and topographical distribution) and function.^[^ ^71^, ^74^ ^]^ The non‐keratinized epithelium (JE and SE), characterized by basal, intermediate, and superficial layers, lines the wall of the gingival sulcus, a physical space between the tooth and the gingiva and home of the gingival microbiota.^[^ ^71^, ^75^ ^]^ Anatomically, the JE connects the gingiva to the tooth, with a multilayered semipermeable barrier to allow the transport of macromolecules, host factors (e.g., cytokines, plasma proteins or immunoglobulins) and polymorphonuclear leukocytes (neutrophils) infiltration, essential for the maintenance of tissue homeostasis (Table 2).^[^ ^71^, ^73^, ^74^, ^75^ ^]^ At the sulcus, the JE shifts to SE, identified as a transitional epithelium between JE and OGE.^[^ ^76^ ^]^ Due to proximity to the gingival microbiota, the architectural stratification and differentiation at SE is greater than JE, resulting in a permeable, but resilient, barrier to withstand microbial challenge.^[^ ^76^ ^]^ The OGE covers the remaining margin of the gingiva and is composed of four strata (basale, spinosum, granulosum, and corneum),^[^ ^71^, ^73^ ^]^ in which the cellular organization leads to the formation of a barrier resistant to external factors, pathogens, or mechanical forces (e.g., mastication or shear).^[^ ^71^, ^73^ ^]^ Keratinocyte cells reside within the gingival epithelium (JE, SE, and OGE).^[^ ^71b^ ^]^ As principal constituents of the gingival barrier, these cells undergo biochemical and morphological changes during their differentiation and migration throughout the layers, aided by cytokeratin proteins, that are specific across epithelia and contribute to the barrier structural integrity to withstand mechanical stress (reviewed in^[^ ^71^, ^73^, ^74^, ^76^ ^]^). The cyto‐anatomical architecture of the gingival epithelium (JE, SE, and OGE) is supported by the underlying connective tissue (lamina propria), mainly composed of collagen type I and III organized into bundles.^[^ ^73^, ^74^, ^77^ ^]^ The connective tissue is rich in stromal cells, blood vessels, nerves, and tissue‐resident immune cells (neutrophils, B‐ and T‐cells, and macrophages) (Table 2).^[^ ^71^ ^]^ Within the gingival lamina propria, the vascular network covers the gingival margin and the junctional epithelium, where tissue surveillance takes place to preserve tissue homeostasis.^[^ ^71^, ^77^ ^]^

#### 3.2.2 Cyto‐Anatomical Architecture of the Gingival Tissue: Microbiota

The gingival microbiota (biomass of 10^12^ – microbes/person) is housed in the gingival sulcus (subgingival microbiota) and, together with the supragingival, tongue dorsal, saliva, and buccal mucosa microbiota, is the second largest microbiota in the human body (Figure 2).^[^ ^1^, ^6^, ^33^, ^71^ ^]^ Compositionally, the gingival microbiota is dominated by five bacterial phyla (*Firmicutes, Actinobacteria, Bacteroidetes, Fusobacteria*, and *Proteobacteria*) in conjunction with fungi and bacterial viruses.^[^ ^78^ ^]^ Although individuals share a similar microbial composition, each person has a unique microbial signature as a result of different bacterial adaptation toward the host (inter‐individual variability), evident also among different dental sites (intra‐variability).^[^ ^16^, ^25^, ^78^, ^79^ ^]^ In fact, anatomical architecture and biological features of the sulcus dictate biofilm composition and organization.^[^ ^80^ ^]^ Local gradients of oxygen, pH, nutrients, and metabolites within the sulcus, in conjunction with host's selective forces (e.g., salivary flow, adhesion proteins, shedding, and host's responses to microbial challenges) and interbacterial dialogues (chemical and physical contact), support the formation and the stability of compositionally diverse ecosystems reflecting different microbial biogeographical arrays.^[^ ^1^, ^2^, ^16^, ^80^, ^81^ ^]^ Micron‐scale biogeographical analysis of dental plaque biofilm has demonstrated that *Streptococcus* and *Actinomyces* species attach to the base of the tooth (anaerobic environment) forming the initial biofilm; *Corynebacterium* species adhere to the formed biofilm and, by protruding filaments toward the periphery (aerobic environment) act as bridging taxa to promote binding of other species (*Streptococcus, Aggregatibacter, Porphyromonas, Neisseriaceae, Fusobacterium, Leptotrichia*, and *Capnocytophaga*) within the biofilm structure.^[^ ^81a^ ^]^ Consequently, the anatomical, physiological, functional, and physical features of the sulcus (host), in combination with the microbe‐microbe communication established within the biofilm, are fundamental traits in dictating the composition and assemblage of the oral microbiota and, thus contributing to tissue homeostasis.

#### 3.2.3 Gingival Host–Microbiota Interactions: Eubiosis and Dysbiosis

Gingival homeostasis is maintained by balanced interactions between the host and its microbiota.^[^ ^1^, ^2^ ^]^ Mutualistic evolution has shaped this interaction by providing a stable ecological niche and the necessary nutrients for the microbiota to adapt and thrive despite host changes and lifestyle habits.^[^ ^1^, ^2^ ^]^ Consequently, the microbiota has formed stable ecosystems that strengthen the gingival barrier, instruct the immune system and participate in sustaining local and systemic health.^[^ ^1^, ^71^ ^]^ At the sulcus, the host tightly controls microbial composition via fitness, adherence, and metabolites.^[^ ^1b^ ^]^ Salivary proteins and enzymes (e.g., mucins, lysozymes, or lactoferrin) facilitate microbial attachment essential for biofilm initiation and stabilization, providing a binding site for commensal bacteria and inhibiting others, or facilitating their elimination.^[^ ^1^, ^82^ ^]^ In addition, the inorganic constituents of saliva (e.g., sodium, chloride, calcium, or magnesium) and shear flow, via epithelium shedding, deliver nutrients to the ecosystem, while regulating the physiological pH of the community and promoting the elimination of pathogenic bacteria anchored to the epithelium.^[^ ^16^, ^82^, ^83^ ^]^ Lastly, the proximity between the epithelium and the microbiota triggers the homeostatic immune response at the sulcus via secretion of host factors collected within the gingival crevicular fluid (GCF) (e.g., cytokines, immunoglobulins, plasma proteins).^[^ ^71a^ ^]^ The permeability of the JE allows the constant influx of neutrophils between the epithelium and the microbiota, where they mediate microbial surveillance through phagocytosis or activation mechanisms, such as degranulation and neutrophil extracellular traps (NETs) formation.^[^ ^71^, ^84^ ^]^ In response to host regulatory mechanisms, the microbiota maintains control over biofilm composition and structure by monitoring nutrients, metabolites, and pH, but also protects the host from infection.^[^ ^1^, ^81^ ^]^ Within the ecosystem, bacteria exhibit a hierarchical organization in which species are classified as primary and secondary colonizers, or consumers and producers.^[^ ^1^, ^2^, ^81^, ^85^ ^]^ For example, *Streptococcus* and *Actinomyces* are primary colonizers of the oral surface favoring the colonization of other species of the gingival margins.^[^ ^2^, ^81^ ^]^ Moreover, at the biofilm periphery *Streptococcus spp*. produces lactate, carbon dioxide, acetate and hydrogen peroxide (H_2_O_2_), by consuming oxygen and sugar. Such byproducts are then available as metabolites for facultative anaerobic species present at the base layers of the biofilm, while inhibiting H_2_O_2_‐susceptible microbes that could potentially cause host infections and disrupt the biofilm assemblage.^[^ ^1^, ^81^ ^]^

Although there are several mechanisms of both host and microbiota to preserve gingival homeostasis, external and internal factors, which are not yet fully defined, could promote imbalanced interactions between host and microbiota in the gingival sulcus leading to the onset of periodontal conditions (e.g., gingivitis or periodontal disease (PD)) (Table 3).^[^ ^1^, ^2^, ^85^ ^]^ Among the periodontal conditions, PD is a chronic inflammation affecting 50% of the U.S. population and causing the disruption of the periodontium.^[^ ^86^ ^]^ PD is currently explained by the polymicrobial synergy and dysbiosis (PSD) model, which attributes the disease to self‐reinforcing polymicrobial dysbiotic interactions (Table 4) within the biofilm and strengthening of host pro‐inflammatory conditions at the sulcus.^[^ ^2^, ^81^, ^85^ ^]^ If left untreated, the sulcus can become a reservoir of pathogenic bacteria potentially leading to periodontal and periodontal‐derived conditions (e.g., Alzheimer's disease, inflammatory bowel disease (IBD), rheumatoid arthritis).^[^ ^1^, ^85^ ^]^ Although simplified, at the center of dysbiotic interactions in the PSD model and in PD is the red complex, a group of pathogens including *Porphyromonas gingivalis*, *Treponema denticola*, and *Tannerella forsythia*, whose relative abundance increases during disease and whose mutually reinforcing interactions contribute greatly to the clinical manifestation of pathological conditions.^[^ ^81b^ ^]^ According to the PSD model, PD is the result of a non‐resolving and tissue‐destructive host response, due to presence of ‘keystone pathogens’ within the pathogenic community. These species are able to modulate the host response in ways that impair immune surveillance and tip the balance from homeostasis to dysbiosis.^[^ ^2^, ^81^, ^85^ ^]^ Specifically, *P. gingivalis* is considered a keystone pathogen that can, at low abundance, establish synergistic relationships with other members of the community (known as *P. gingivalis* interactome), while manipulating host immunity and inflammation to support its persistence.^[^ ^2^, ^81^, ^85^ ^]^ During PD, *P. gingivalis* adheres to gingival epithelial cells and uses complex machinery to evade the host's inflammatory and immune response, while invading and remaining viable within host cells.^[^ ^2^, ^81^ ^]^ *P. gingivalis*: 1) adheres and invades the epithelial cells by transient expression of proteases called gingipains, and cytoskeleton remodeling that facilitates its engulfing and supports its growth; 2) contributes to its persistence in the gingival epithelial cells by enhancing cellular proliferation via regulation of cyclic adenosine monophosphate (cAMP) levels and inducing antiapoptotic programs; 3) regulates negatively the production of H_2_O_2_ and the associated release of IL‐6, which can impair its intracellular persistence in the host cells; 4) subverts the host's immune system by inducing chemokine paralysis (CXCL8, 9, 10, and 11) resulting in not recruitment of neutrophils and T‐helper1 development; 5) produces glycine and thiamine to stimulate the growth of *T. denticola*, which produces metabolite to support the growth of *P. gingivalis*; 6) increases in relative abundance in the sulcus aided by *Fusobacterium nucleatum*, which favors a more anaerobic environment for *P. gingivalis*, facilitating its growth.^[^ ^71^, ^81^ ^]^

Currently, there is no clear consensus on the primary event that leads to dysbiosis, whether a dysbiotic microbiome drives gingival inflammation or is a consequence of enhanced host inflammation.^[^ ^71^, ^85^ ^]^ Moreover, the inability to study the trajectory of disease in pre‐clinical or clinical models, together with the high variability of the microbiota and inflammatory mechanisms in animal models, hinders the ability to predict or identify the original triggers of periodontal conditions.^[^ ^87^ ^]^

#### 3.2.4 In Vitro Modeling of Gingival Host–Microbiota Interactions

Several in vitro systems (Figure 2) have been developed and validated to study the effect of oral microbiota on periodontal health and disease (Table 5). The gingival cyto‐anatomical architecture was mimicked by culturing, in two‐ or three‐dimensional configurations, primary human gingival keratinocytes and stromal cells isolated from tissue biopsies (primary or immortalized cell lines).^[^ ^88^ ^]^ Layering and differentiation of the epithelium were achieved by seeding keratinocytes directly onto porous membranes and cultured at the air‐liquid interface.^[^ ^88a^ ^]^ Additional strategies co‐cultured epithelium and stroma on biopolymers substrates, largely collagen‐based hydrogels, for replicating the organizational complexity of the gingiva. These models were based on epithelial cells grown on top of stromal cells embedded in a type I collagen hydrogel cultured on tissue inserts at an air‐liquid interface.^[^ ^19^, ^88^, ^89^ ^]^ To overcome gel structural instability,^[^ ^19a^ ^]^ structural proteins, such as collagen and silk, have been used with the purpose of engineering materials that can bridge the biotic/abiotic interface replicating oral mucosa in planar or anatomical geometries.^[^ ^10^, ^25^ ^]^ Blending silk with collagen can retain the biological cues of collagen, while increasing the mechanical capabilities to mitigate the cell‐mediated remodeling in collagen matrices and providing long‐term structural stability.^[^ ^90^ ^]^ These culture methods provide gingival‐like morphological features, such as cellular organization in multi‐layer, proliferative‐differentiated epithelia (Ki67), differentiation (E‐Cadherin or Keratin), gingival sulcus geometry and physical properties, and response to pathogens.^[^ ^10^, ^19^, ^88^, ^89^ ^]^ Enhancement of the cyto‐anatomical properties of the oral mucosa (e.g., proliferation, differentiation and stratification, and barrier functions) were achieved through oral‐on‐a‐chip^[^ ^10^, ^26^, ^91^ ^]^ and physiologically relevant bioreactors,^[^ ^25^, ^92^ ^]^ to mimic the oral mechanical conditions, such as mucosa shedding, removal of pathogenic bacteria and buffering actions of the oral macroenvironment.^[^ ^2^, ^81^ ^]^ In addition, these physiologically relevant dynamic platforms allowed to profile GCF interstitial flow^[^ ^10e^ ^]^ or incorporate artificial saliva formulation and shear dynamics.^[^ ^25c,d^ ^]^ This proof‐of‐principle GCF‐on‐a‐chip fabricated via lithography will further advance the current understanding of physiological release of inflammatory markers, which are indicative of periodontal health conditions.^[^ ^2c^ ^]^ Moreover, by recreating tissue architecture and physiological salivary shear composition and mechanism stress, these models can be used as a platform to investigate long‐term host–microbiota imbalances.^[^ ^25^, ^92^ ^]^ Investigation of healthy tissue‐microbiota interactions employed patient‐derived microbiota, from salivary or subgingival plaque, co‐cultured in an collagen‐based oral mucosal tissue equivalent^[^ ^89^ ^]^ and fully anatomical silk‐collagen scaffolds.^[^ ^10^, ^25^ ^]^ Microbial interactions have been implemented with different approaches, by employing single species or artificial biofilms developed from bacterial lines and exposed directly to the epithelium^[^ ^88a,b^ ^]^ or embedded in gelatin discs and then exposed to the epithelium.^[^ ^88^, ^92^, ^93^ ^]^ These studies focused on the host's barrier properties and cytokine profiles as well as biofilm viability, integrity, and abundance. Furthermore, given the interdependency between host behavior and oral microbiota in preserving tissue homeostasis,^[^ ^1^, ^71^ ^]^ oral mucosa culture systems have incorporated monocytes or peripheral blood mononuclear cells to study the innate immune response in the periodontal pocket, key in the establishment of periodontal conditions.^[^ ^92^, ^93^ ^]^ Functional host–microbiota readouts focused on the preservation of host cyto‐anatomical architecture (e.g., stratification, differentiation, and barrier properties),^[^ ^10^, ^25^, ^88^, ^89^ ^]^ as well as cytotoxicity, viability,^[^ ^10^, ^25^, ^93^ ^]^ and secreted factors^[^ ^10^, ^25^, ^88^, ^89^, ^92^, ^94^ ^]^ in response to microbial challenge. Microbiota analysis, instead, centered on viability,^[^ ^10^, ^25^, ^88^, ^89^ ^]^ composition and richness (16S rRNA gene sequencing) in light of a diverse oxygen‐tolerance by bacterial species within the microbiota,^[^ ^10^, ^25^, ^92^ ^]^ or invasion in periodontal conditions.^[^ ^88b^ ^]^ Alternative strategies have also used oral mucosa organoids to test oral mucosa herpes simplex or papillomavirus infections^[^ ^95^ ^]^ or commercially available oral mucosa constructs, such as EpiOral or EpiGingival (MatTek Corporation)^[^ ^88^, ^94^ ^]^ to test commercial oral care products.^[^ ^96^ ^]^

#### 3.2.5 Emerging Clinical Strategies Targeting Gingival Host–Microbiota Interactions in Disease States

Mimicking host–microbiota gingival interactions and the transition between disease states opens the door to using in vitro models to validate novel therapeutics in anticipation of clinical trials if physiological relevance is ensured.^[^ ^97^ ^]^ Some in vitro models leverage multi‐omics analyses, such as host biomarker profiling and the relative abundance of microbiota taxa, to underscore the mucosa's inflammation state and the associated microbiota profile (16S rRNA gene sequencing).^[^ ^97^, ^98^ ^]^ Although these methodologies have been explored, there is a need to develop precision medicine approaches due to the high degree of individual genetic diversity, microbiota variability, and cultural and lifestyle factors that affect oral health status, as oral conditions are highly multifactorial and highly dynamic over time.^[^ ^97^, ^99^ ^]^ This approach would support further investigation of specific mechanisms by which therapeutics affect oral health via predictive validation, design of personalized medicine approaches, and, ultimately, standardization of diagnostic criteria via in vitro modeling.

Precision medicine therapeutics for oral health diseases encompass a range of innovative treatments, such as probiotics and specialized pro‐resolving lipid mediators (SPMs), which can be tailored to individual patient profiles to target disease mechanisms more effectively and highlight the potential breakthroughs in managing conditions like periodontitis or other oral health disorders.^[^ ^86^, ^99^, ^100^ ^]^ Although not yet fully defined, the mechanism of action of probiotics appears to be the environmental pH modulation through the production of antibacterial peptides (bacteriocins) and reactive oxygen species (ROS), as well as the ability to out‐compete with pathogenic microorganisms for colonization and growth within the ecosystem, interfering with their metabolism and living conditions (nutrients – sugars, amino acids, or vitamins – and adherence).^[^ ^100b–d,g–i^ ^]^ Interspecies interactions can have varying effects on specific microbes, such as inhibition or predation, that can be leveraged to restore the balance between microbial communities^[^ ^100b–d,g–i^ ^]^ This mechanism has been elucidated for selected probiotic species: *Lactobacillus plantarum*, with superior inhibitory control over *Candida albicans* and *Streptococcus mutans* growth, agents of dental caries, compared with other *Lactobacillus* strains (*plantarum* ATCC 8014, *plantarum* ATCC 14917, and *salivarius* ATCC 11741).^[^ ^101^ ^]^ To date, no probiotic products have been FDA‐approved as live biotherapeutics. They are strictly sold as supplements, which do not mandate proof of efficacy, and they are safe to use for long‐term consumption in children and adults.^[^ ^100^, ^102^ ^]^ In this context, in vitro models would contribute to elucidating how different probiotic strains affect pathogenic species' relative abundance within the oral microbiota, and designing customized probiotic treatments targeting patient's specific pathogens, which will lead to personalized treatments targeting specific pathogens leveraging ad hoc delivery (e.g., lozenge, mouthwash).^[^ ^99^, ^100^ ^]^ Other examples of developing precision medicine approaches for oral health diseases include SPMs, such as lipoxins and resolvins, which are known to regulate host responses and promote the resolution of inflammation.^[^ ^86^, ^99^, ^100^ ^]^ SPMs target a broad spectrum of cell types (e.g., neutrophils, macrophages, dendritic cells, and NK cells) by interacting with cell receptors to mitigate inflammation and simultaneously facilitate tissue repair.^[^ ^86^, ^99^, ^100^ ^]^ SPMs have shown several positive effects, both in vitro and in vivo, including the increased resistance of mesenchymal stem cells to apoptosis, the regeneration of periodontal tissues, and protection from osteoclast‐mediated bone destruction, as demonstrated by clinical trial data (phase 1‐ methyl ester‐benzo‐lipoxin A4 (BLXA_4_)).^[^ ^86^, ^99^, ^100^ ^]^ SPMs have also elucidated a new understanding of the etiopathology of periodontal disease and, thus, how it should be treated.^[^ ^97^, ^99^ ^]^ Modulating the body's immune response with SPMs may not only halt disease progression but also promote the resolution of inflammation and tissue repair, shifting the focus from merely controlling microbial factors to restoring immune balance and protecting host tissues.^[^ ^10^, ^25^, ^97^, ^99^, ^102^, ^103^ ^]^

Commercially available 3D in vitro models of the buccal and gingival mucosa, EpiOral™ and EpiGingival™ (MatTek), have established protocols for toxicological studies of novel therapeutics (such as SPMs and probiotics) on pre‐selected single or multi‐strain cultures.^[^ ^97^, ^104^ ^]^ For example, the EpiOral™ and the EpiGingival™ models have been employed to evaluate an antimicrobial peptide mouth rinse targeting *Streptococcus mutans* clinical isolate UA140, associated with the onset of dental caries.^[^ ^105^ ^]^ The development of standardized protocols to consider the complexity of oral microbiota will improve the physiological relevance of commercially available models. Additionally, the commercial availability of these models will support standardized and reproducible research efforts.^[^ ^97^, ^104^, ^105^ ^]^

Taken together, probiotics and SPMs, represent a promising platform to revolutionize oral healthcare through personalized medicine, with advanced in vitro models as pivotal tools for developing tailored treatment regimens (i.e., bioreactors and oral‐on‐a‐chips).^[^ ^10^, ^25^ ^]^ Beyond disease conditions affecting the gingiva, other oral health disease indications related to microbial dysbiosis that can also be studied in vitro to develop targeted therapies include dental caries, oral candidiasis, halitosis, peri‐implantitis, oral lichen planus, recurrent aphthous stomatitis, and oral cancers.^[^ ^97^, ^100^ ^]^

### 3.3 Intestinal Tissue

#### 3.3.1 Cyto‐Anatomical Architecture of the Intestinal Tissue: Host

**Figure 3:**
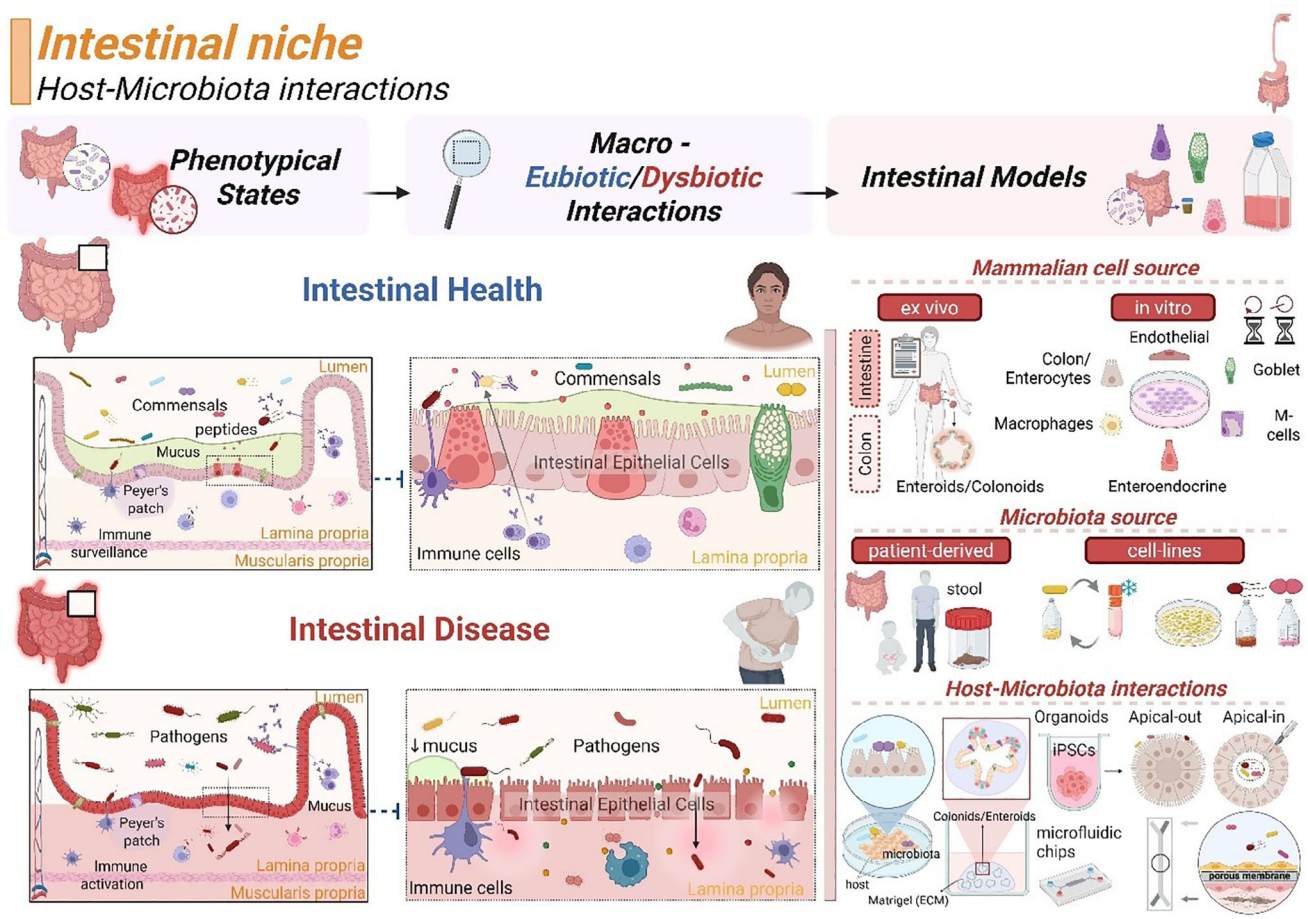
Intestinal niche. Left – Representation of the intestinal niche in healthy and disease (Inflammatory Bowel Disease) states. Right – Overview of the mammalian and microbiota sources employed in current in vitro strategies. Created in BioRender. Ghezzi, C. (2025) https://BioRender.com/ny32e11.

The gastrointestinal tract (GIT) (**Figure** **3**) represents an important junction in the human body due to its bidirectional communication with other tissues, including the oral cavity, brain, and skin, thus, playing a crucial role in overall human health.^[^ ^1^, ^4^, ^106^ ^]^ In addition to the water and nutrient absorption and digestion, as well as waste elimination from the human body, the tissue architecture of the GIT represents a unique barrier against antigens, toxins, and harmful pathogens.^[^ ^107^ ^]^ The GIT includes the esophagus, stomach, and intestine, the latter comprising the small intestine ((Sm‐Int) – duodenum, jejunum, and ileum) and large intestine ((Lg‐Int) – caecum, colon, and rectum), and discussed in this review.^[^ ^3^, ^108^ ^]^ The intestinal cyto‐architecture (Table 1) comprises four layers: mucosa, submucosa, muscularis propria, and serosa.^[^ ^108^, ^109^ ^]^ The mucosa, composed of epithelium, lamina propria, and muscular mucosae, is home to the intestinal microbiota.^[^ ^3^, ^108^, ^109^ ^]^ Although in the intestine most phenotypical traits are shared, Sm‐Int and Lg‐int exhibit some cyto‐architectural differences in the mucosa.^[^ ^3d^ ^]^ Intestinal epithelium has a distinctive architecture composed of tubular invaginations (crypts) and projections to the lumen (villi), the latter absent in the Lg‐Int, and is heterogeneous in cellular composition.^[^ ^3^, ^110^ ^]^ The cytoarchitecture of the crypt‐villi axis forms a continuous, polarized monolayer of proliferative and differentiated intestinal epithelial cells (IECs), each performing specific functions, that overall separate the intestinal lumen from the lamina propria.^[^ ^107^, ^108^, ^110^ ^]^ Pluripotent intestinal stem cells reside at the base of the crypts and undergo proliferation (Transit Amplifying cells – TACs) and differentiation into subpopulations of IECs: secretory – paneth, goblet, tuft and enteroendocrine cells; absorptive – enterocytes, colonocytes and, microfold (M) cells. Although the detailed functions of each population of IECs have been extensively reviewed in,^[^ ^3^, ^108^, ^110^ ^]^ it is important to highlight the functions of enterocytes (Sm‐Int) or colonocytes (Lg‐Int), which are responsible for the intestinal barrier and absorption of nutrients and water, and goblet cells, which are responsible for secretion of mucus for colonization of the microbiota.

Once in the villus, IECs undergo terminal polarization and differentiation, characterized by a brush border of microvilli, central in cellular uptake between the intestinal lumen and epithelium.^[^ ^108^ ^]^ Above the epithelium, protective mucus provides a physical barrier between the intestinal lumen and the epithelium for microorganisms and toxins, while supporting commensal microbial colonization, providing nutrients for bacteria growth.^[^ ^3^, ^108^, ^110^ ^]^ The mucus is mainly characterized by O‐glycosylated mucin‐2 proteins, secreted by goblet cells, with a topographical variation in thickness throughout the intestine. The Sm‐Int presents a single layer of mucus, while the Lg‐Int features two layers, namely an outer layer, which favors commensal bacteria colonization, and an inner, dense layer, that protects the epithelium and is impenetrable to bacteria.^[^ ^3^, ^107^, ^108^, ^110^ ^]^ The intestinal epithelium is surrounded by the lamina propria, comprising stroma, mesenchymal cells, neurons, and vasculature.^[^ ^109^ ^]^ The stroma is characterized by connective tissue rich in extracellular matrix (ECM) composed of collagen type IV, fibronectin, and laminin.^[^ ^108^, ^109^ ^]^ Fibroblast and myofibroblast cells reside in the stroma and are essential in ECM remodeling and regulation of epithelial homeostasis.^[^ ^108^, ^109^ ^]^ The lamina propria is also home to tissue‐resident immune cells (Table 2) (i.e., monocytes, macrophages, B‐ and T‐lymphocytes, basophils, dendritic cells, and mast cells), responsible for tissue homeostasis and antipathogenic response, by probing commensals/pathogens through direct contact between epithelial cells and segmented filamentous bacteria.^[^ ^3^, ^110^ ^]^ Lastly, the submucosa includes vasculature, both blood and lymphatic, lymphoid aggregates (Peyer's plaques in the Sm‐Int), and the enteric nervous system (ENS), important for motility, peristalsis, and communication with the brain (intestine‐brain axis) (reviewed in in^[^ ^108^, ^109^, ^110^ ^]^).

#### 3.3.2 Cyto‐Anatomical Architecture of the Intestinal Tissue: Microbiota

The GIT harbors the largest microbiota in the human body, in both richness and diversity, including bacteria, bacterial viruses, fungi, archaea, and eukaryotic parasites (Figure 3).^[^ ^33^, ^111^ ^]^ Regional biogeographic characteristics govern the intestinal niche, with progressive increase in bacterial load from Sm‐Int to Lg‐Int.^[^ ^3^, ^111^ ^]^ The Sm‐Int harbors a less diverse microbiota (biomass of 10^7^–10^11^ (microbes/person), dominant in *Pseudomonadota* (*Neisserriaceae, Enterobacteriacae, Nitrobacteraceae, Pseudomocadaceae) Streptococcaceae, Prevotellaceae*, *Actinomycetaceae, Veillonellaceae* and *Lactobacillaceae*) in comparison to the Lg‐Int, which hosts a both diverse and rich microbiota (biomass of 10^14^ (microbes/person), dominant in *Bacteroida (Bacteroidaceae), Enterobacteriacae, Enterococcaceae, Veillonellaceae, Bidifobacteriaceae, Lachnospiraceae* and *Ruminococcaceae*).^[^ ^3^, ^28^, ^111^ ^]^ Although a core of bacteria taxa defines the overall intestinal microbiota, differences in microbial composition among individuals are largely influenced by dietary habits, which affect intestinal tissue physiological functions and, consequently, its interactions with the microbiota.^[^ ^2^, ^112^ ^]^ Nevertheless, the population forming the intestinal microbiota can be considered generally consistent among individuals.^[^ ^3^, ^28^, ^111^, ^112^ ^]^ Microbiota composition and relative interactions with the host are finely regulated by physical and biochemical gradients, including pH, O_2_, antimicrobial peptides, nutrients, mucus, peristalsis, and flow velocity.^[^ ^3^, ^28^ ^]^ Macro‐scale biogeographical analysis of the intestinal samples has reported heterogeneity of microbial ecosystems influenced by intestinal macro and microenvironmental conditions.^[^ ^3^, ^28^ ^]^ The Sm‐Int (duodenum) has a rather acidic pH (6) and represents an aerobic environment, features that are essential to prevent pathogen colonization.^[^ ^113^ ^]^ The jejunum and ileum (Sm‐Int) are instead characterized by a more basic (7.5) and anaerobic environment, enabling facultative anaerobic species to thrive.^[^ ^113^ ^]^ Oxygen gradients in the Sm‐Int are promoted by the vasculature underlying the epithelium, resulting in a less oxygenated environment towards the lumen.^[^ ^3^, ^111^ ^]^ Approaching the Lg‐Int (cecum), changes in physical conditions are represented by a decrease in pH (6.3), an anaerobic environment in the lumen, and a slower food transit time than in the Sm‐Int, that leads to greater nutrients availability, resulting in abundance in bacterial colonization.^[^ ^28^, ^113^ ^]^ In the colon (Lg‐Int), the pH increases (6.5‐7) and the microenvironment becomes strongly anaerobic; notably, due to a slower food transit time and the presence of thicker mucus covering the epithelium, the availability of nutrients favors bacterial colonization, particularly of *Bacteriodaceae*, involved in the degradation of polysaccharides.^[^ ^3^, ^28^, ^108^, ^110^, ^113^ ^]^ Consequently, the cyto‐anatomical and physical features of the host are fundamental traits in dictating diversity, richness, and biogeography of the intestinal microbiota and, thus affecting tissue homeostasis.

#### 3.3.3 Intestinal Host–Microbiota Interactions: Eubiosis and Dysbiosis

Intestinal homeostasis is maintained by balanced host–microbiota interactions across different regions of the intestine.^[^ ^110b^ ^]^ Cyto‐anatomical architecture, mucus organization, immune system activation in combination with host's lifestyle habits, diet, and age influence microbiota intestinal topography and functions.^[^ ^3^, ^28^, ^108^, ^110^, ^111^ ^]^ On the crypt‐villus axis, IECs form a semipermeable physical and biochemical barrier that separates the microbiota from the immune system, while minimizing physical interactions between the microbiota and the epithelium through mucus layers.^[^ ^33^, ^110^, ^114^ ^]^ At the barrier, goblet cells are responsible for mucus production (MUC2), which can be constitutively synthesized to impose bacterial segregation from the epithelium, or regulated upon damage to the epithelium by harmful agents.^[^ ^114^, ^115^ ^]^ In the mucus layers, IECs (enterocytes, goblet, and Paneth cells) release a wide range of peptides (e.g., anti‐microbial peptides, lysozyme, cathelicidins), which hinder bacterial colonization at the epithelium and pathogen invasion.^[^ ^110a^ ^]^ Interestingly, commensal bacterial cells protect epithelial cells from exposure to pathogens by using their mucin‐binding site via adhesin proteins to trap pathogens in the mucus structure.^[^ ^114^ ^]^ As a first line of defense, intestinal barrier is also reinforced by production of cell‐derived products (e.g., Trefoil factor 3 or Resistin‐like molecule‐β), whose function is to stabilize structural mucus integrity via mucin crosslinking, to promote IECs repair and migration, and to regulate host's response during inflammation.^[^ ^110a^ ^]^ Therefore, structural integrity and the thickness of the mucus play a central role in the mechanisms governing intestinal health.^[^ ^107^ ^]^ At the mucus interface, immune cells together with IECs secrete immunoglobulins and perform antigen/bacteria priming, which collectively determine the colonization and organization of commensals, as well as the regulation of the intestinal epithelial barrier.^[^ ^3^, ^28^, ^107^, ^110^, ^114^, ^115^ ^]^ Commensal and pathogen recognition is finely tuned by IECs immunoregulation and by the adaptive immune response at specific intestinal sites or by signaling mechanisms, such as Peyer's patches, segmented filamentous bacteria (SFB) and Toll‐like receptors (TLRs).^[^ ^3^, ^110^ ^]^ At the Peyer's patches, the only intestinal site where the epithelium and bacteria are in physical contact, M‐cells sample luminal antigens or intact bacteria and, subsequently, transfer them directly to the underlying immune cells (dendritic cells or lymphocytes) for pattern recognition.^[^ ^3^, ^110^ ^]^ Priming and bacterial recognition can also happen via SFB, gram‐positive bacteria bound to IECs and critical in stimulating mucosal Th17 cell differentiation.^[^ ^116^ ^]^ Lastly, TLRs are widely expressed on the apical and basolateral membrane of polarized IECs, contributing to their sentinel role for commensals/pathogens and activating their immunoregulatory mechanisms.^[^ ^110^, ^117^ ^]^ Expression of TLRs on IECs is instrumental in recognizing microbial‐associated molecular patterns (MAMPs), which regulate commensals mucosal tolerance, anti‐microbial peptide suppression, and bacterial colonization, as for the Polysaccharide A (PSA), a MAMPs of the commensal bacteria *Bacteroides fragilis*.^[^ ^117^ ^]^ Secretion of MAMPs and activation of TLRs stimulate production by B‐cells in the lamina propria of immunoglobulin A (IgA), which is later translocated into the lumen by IECs via transcytosis as secretory immunoglobulin A (SIgA); in the lumen, SIgA regulates the composition and function of the microbiota by restricting its access to the epithelium, in case of pathogens, or stimulating biofilm formation for commensals.^[^ ^28^, ^107^, ^118^ ^]^

As the intestine is majorly involved in nutrient absorption and metabolic processes, microbiota does regulate the host's metabolism.^[^ ^110^, ^111^ ^]^ For example, obligate anaerobes, such as *Clostridium clusters IV* and *IXa*, *Faecalibacterium prausnitzii*, and *Bacteroides thetaiotaomicron*, have been shown to be involved in breaking down carbohydrates into short‐chain fatty acids (SCFA), such as acetate and butyrate.^[^ ^114^, ^119^ ^]^ Butyrate, a fiber fermentation product of intestinal microbiota, promotes Mucin‐2 (MUC2) transcription, favoring mucus production and secretion, as well as inhibiting inflammation and tumor growth.^[^ ^114^ ^]^ Specifically, butyrate regulates immune cells in the mucosa by enhancing the activation of regulatory T‐cells (Treg) and inhibiting over activation of macrophages, dendritic cells or neutrophils.^[^ ^120^ ^]^ Moreover, colonocytes in the crypts use butyrate as part of the metabolism to protect stem cells from exposure to this metabolite, known to hinder their ability to proliferate, and therefore acting as a metabolic barrier.^[^ ^114^, ^119^ ^]^

Overall, intestinal cells exert several functions that result in a dynamic barrier that protects the host from pathogen invasion and infection.^[^ ^3^, ^107^, ^110^, ^114^ ^]^ However, microbial dysbiosis and impairment of the epithelial barrier can lead to a range of gastrointestinal disorders (e.g., IBD or colorectal cancer) (Figure 3).^[^ ^28^, ^111^ ^]^ IBD is a chronic inflammatory condition comprising two syndromes: Crohn's disease (CD), affecting the entire GIT, and Ulcerative Colitis (UC), restricted to the Lg‐Int.^[^ ^28^, ^107^ ^]^ 1.3% of the US adult population suffers from IBD and symptoms range from GIT complications to abdominal pain, and weight loss.^[^ ^121^ ^]^ Although genetic and environmental factors, such as smoking, diet, and medications, may contribute to the clinical manifestation of the disease, key features of IBD are intestinal epithelium dysfunction, variations in microbial diversity (dysbiosis), and dysregulation of innate and adaptive immune systems.^[^ ^28^, ^107^, ^120^, ^122^ ^]^ Following exposure to environmental stimuli, the mechanisms of IBD consist of: 1) epithelial dysfunction characterized by disruption of barrier integrity and increased permeability; 2) altered functions of IECs (goblet and Paneth cells) related to defense mechanisms, such as mucus thickness and antimicrobial activities; 3) increased entry of luminal bacteria or pathogens; and 4) innate and adaptive activation leading to the release of pro‐inflammatory factors and of primed immune cells in the bloodstream, which may contribute to the perpetuation of intestinal inflammation.^[^ ^120^, ^122^ ^]^ Phenotypical changes at the intestinal barrier include downregulation of barrier junctional proteins, such as e‐cadherin, occludin, and claudin 5,8, determining reduction in cell‐cell contacts, increased intestinal permeability (up to 40–50%) and consequent impairment of barrier integrity.^[^ ^120^, ^122^, ^123^ ^]^ Compromised barrier integrity is also associated with alterations in the mesh‐like structure of the mucus layers.^[^ ^120^, ^122^ ^]^ Elevated synthesis of truncated O‐glycan structures by goblet cells results in limited crosslinking of MUC2, failure in the stabilization of the thicker mucus layers, as well as reduced viscoelastic properties and barrier functions.^[^ ^3^, ^107^ ^]^ Patients affected by UC present a similar phenotype, although a decrease in goblet cell density results in lower mucus production and secretion, hence in a thin mucus layer.^[^ ^107^ ^]^ Further IECs’ dysfunctions feature Paneth cells, dendritic cells, and macrophages by dampening the communication between IECs and immune cells (dendritic to T‐cells), as in the case of antimicrobial mechanisms associated with Nucleotide‐binding oligomerization domains 2 (NOD2) and autophagy.^[^ ^120^, ^122^, ^124^ ^]^ Lastly, a common trait in IBD patients is immune activation.^[^ ^122a^ ^]^ In homeostatic conditions, dendritic cells, macrophages, neutrophils, and lymphocytes represent the first line of defense to prevent pathogen colonization.^[^ ^122a^ ^]^ Antigen presentation via dendritic cells and macrophage activation, host's response, and microbiota metabolism are crucial in promoting regulation, differentiation, and activation of T‐cells (Treg and Th1, 2 or 17).^[^ ^120^, ^122^, ^124^ ^]^ In IBD patients, increased activation and decreased Treg compromise the Th17/Treg ratio, leading to cumulative mucosal T‐cell response across the different lineages, and augmented circulation of primed lymphocytes, contributing to chronic inflammation.^[^ ^120^, ^122^, ^124^ ^]^ Along with uncontrolled inflammation, IBD is also related to altered microbiota composition (Table 3).^[^ ^28a^ ^]^ Biopsy samples of IBD patients present elevated concentration of dysbiotic bacteria at the mucosal surfaces (Table 4), characterized by overall decreased microbial diversity especially in *Firmicutes* (*Clostridium species*) and increased relative abundance of *Proteobacteria, Bacteroidetes*, and *Enterocacteriaceae*.^[^ ^28^, ^120^, ^122^, ^123^, ^125^ ^]^ As dysbiosis progresses, unbalanced interactions between host and microbiota result in altered metabolic processes, for example, SCFA, tryptophan or bile acids.^[^ ^120^, ^122^ ^]^ As previously mentioned, SCFAs are pivotal in regulating mucosal immunity (B‐ and T‐cells).^[^ ^114^, ^120^ ^]^ Decreased metabolism of SCFAs promotes the growth of butyrate‐producing bacteria such as *Escherichia coli*, as well as M1 macrophage polarization and associated release of pro‐inflammatory cytokines enhancing inflammation.^[^ ^126^ ^]^ Moreover, compromised tryptophan metabolism by host (pathway: casein and serotonin) and bacteria (pathway: indole) enhances IBD symptoms due to failed inhibition of pro‐inflammatory cytokines release.^[^ ^120^ ^]^

Despite considerable progress in intestine processes, causality in the onset and progression of IBD has yet to be understood. Studies have shown a correlation between the role of the microbiota and over‐activation of the immune response, along with environmental factors and diet, that could be determinants in IBD progression.^[^ ^122^, ^127^ ^]^ Several directions are being pursued to investigate host–microbiota interactions in healthy and disease states and to identify mechanisms of communication during pathogenesis.^[^ ^120^, ^122^, ^128^ ^]^ Moreover, given the complexity of the intestinal microbiota, the identification of single bacteria species driving disease or the response to the bacteria to environmental changes are still a challenge.^[^ ^33^ ^]^ Similarly, differences in microbiota composition, immune and metabolic regulation, together with the lack of established and standardized study protocols, contribute to their limited human relevance, opening the door to the development of human‐based physiologically relevant in vitro models as a suitable approach to study complex host–microbiota interactions.^[^ ^128^, ^129^ ^]^

#### 3.3.4 In Vitro Modeling of Intestinal Host–Microbiota Interactions

To advance research on intestine‐microbiota interactions, several tissue engineering strategies (Figure 3) have been explored to recapitulate the complex cyto‐anatomical architecture displayed in the intestine (Table 5).^[^ ^10^, ^130^ ^]^ Features of the intestine and cellular populations were emulated as monocultures or co‐cultures grown in two‐ and three‐dimensional approaches (e.g., submerged, Transwell inserts, embedded in collagen‐coated scaffold constructs or other matrices).^[^ ^10^, ^128^, ^130^ ^]^ Immortalized cell lines derived from human colon adenocarcinoma (Caco‐2 or HT‐29) have been extensively used to display characteristics of mature enterocytes, such as epithelium polarity, brush border, and villin expression, although they are limiting in replicating intestinal functions (e.g., hormone secretion, mucus or hydrolases).^[^ ^10^, ^128^, ^130^ ^]^ While monocultures of immortalized intestinal epithelial cells have been instrumental in permeability studies, co‐cultures of immortalized enterocytes with fibroblasts or endothelial cells have enhanced intestinal epithelium cyto‐anatomical and functional features (brush border, villi, enhanced polarization, barrier, and permeability).^[^ ^10^, ^130^, ^131^ ^]^ Given the pivotal role of immune sensing and activation in preservation of intestinal homeostasis, it has been reported that intestinal co‐cultures can influence the differentiation of enterocyte‐like cells into immune cells, as in the case of Caco‐2 cells in M cells, when co‐cultured with Peyer's patch lymphocytes.^[^ ^128^, ^132^ ^]^ Despite their limitations, immortalized cell lines provide major advantages in comparison to primary cells, which have low survival, and for some cell populations (e.g., M cells) their abundance is scarce.^[^ ^128^ ^]^ Other strategies have also relied on 2D organoids monolayers (enteroids or colonoids) derived from human donors or iPSCs.^[^ ^128^, ^129^, ^130^ ^]^ Prior to differentiation, stems cells collected from human donors are being dissociated, embedded in ECM (commonly Matrigel), plated on Transwell inserts, and differentiated to enteroids or colonoids.^[^ ^130^, ^133^ ^]^ A major advantage of organoids is the cultivation of anaerobic bacteria, which are microinjected into the hypoxic lumen (apical in) of the organoid; this culture methodology allows studying certain aspects of intestinal microbial communities and pathogenesis (barrier, metabolism, composition, nutrients).^[^ ^134^ ^]^ Additionally, a more physiologically relevant epithelium‐microbiota interactions can be studied using organoids with reversed polarity (apical out) that facilitate co‐culture with bacteria on the outer side, thus avoiding the need for microinjections.^[^ ^15^, ^130^, ^135^ ^]^ The replication of three‐dimensional cyto‐anatomical architecture and the associated gradients have been also achieved by means of scaffolding techniques, which can be tailored for personalized medicine.^[^ ^109^ ^]^ Synthetic polymers (PLA/PLGA), or natural polymers, such as ECM‐derived components (e.g., collagen, elastin, or fibrin or hyaluronic acid) or silk fibroin, have replicated the cell‐to‐cell interactions, as well as the native anatomical organization of the intestine (crypt‐villus axis) achieved by combining organoids cultures and tissue engineering procedures.^[^ ^109^, ^130^, ^136^ ^]^ The advantages of these systems are they can be co‐culture with several cellular populations, such as endothelial cells, fibroblasts, myofibroblasts or immune cells.^[^ ^10^, ^130^, ^137^ ^]^ Lastly, commercially available intestinal models (EpiIntestinal^TM^) were developed to study drug absorption, efficacy, and toxicity.^[^ ^138^ ^]^ Collectively, these in vitro models have successfully replicated different epithelial populations (enterocytes, goblet cells, Paneth cells or enteroendocrine cells), intestinal phenotypic traits (polarity, villi, and mucins), expression of junctional markers (ZO‐1, Occludin‐1, Claudin‐4, VE‐Cadherin), barrier properties (permeability) and response to pathogens.

Given the role of oxygen gradients in shaping intestinal biogeography, traditional 2D cultures have been adapted to sustain aerobic‐to‐anoxic environments, which is necessary to fully replicate host–microbiota interactions.^[^ ^130^, ^133^, ^135^ ^]^ Microbial populations consisted of selective bacterial lines (e.g., *E. coli, Bacteroides fragilis*, probiotics *– Bifidobacterium longum* or *Lactobacillus reuteri*) or microbiomes derived from feces and co‐cultured with host cells in static or dynamic (microfluidic or bioreactor systems) conditions to represent intestinal mechanical stimulation (flow and peristalsis).^[^ ^10^, ^130^, ^133^ ^]^ These models enabled the culture of intestinal epithelial cells (terminally differentiated or organoids) coupled with bacterial cells, fostering anoxic environments to support anaerobic bacteria, mimicking the environment and physiological interactions, and thus facilitating intervention methods (i.e., pre/pro‐biotic, fecal transplants, or pharmacological tests) aimed at modulating microbiota‐derived diseases.^[^ ^10^, ^139^ ^]^ Ultimately, considering the significant role of the mucus layer in the intestine, in vitro strategies have modeled the composition and properties of mucus using mucus‐secreting cells (Caco‐2), commercially available gastric mucins or mucin substitutes (alginate, polyethylene glycol‐PEG or fluoride‐assisted mucus surrogate). These mucus models have been used to study bacterial colonization, bacterio‐mucus interactions, and drug discovery.^[^ ^140^ ^]^

Overall, host–microbiota readouts focused on intestinal cellular population, architecture, and viability (polarity, barrier properties, mucus, and microvilli),^[^ ^10^, ^15^, ^130^, ^133^, ^139^ ^]^ microbial colonization, composition, and invasion,^[^ ^10^, ^15^, ^133^, ^139^ ^]^ oxygen gradients,^[^ ^10^, ^133^, ^139^ ^]^ and anti‐bacterial response.^[^ ^130b^ ^]^

#### 3.3.5 Emerging Clinical Strategies Targeting Intestinal Host–Microbiota Interactions in Disease States

Current in vitro models to house intestinal microbiota have emerged as promising tools for developing precision medicine strategies for various diseases. A leading commercially available product is EpiIntestinal^TM^, a tissue model of the small intestine (MatTek).^[^ ^138^, ^141^ ^]^ The EpiIntestinal^TM^ SMI‐100 models differentiated intestinal epithelial cells (enterocytes, Paneth cells, M cells, Tuft cells, and intestinal stem cells) and the underlying lamina propria.^[^ ^138^, ^141^ ^]^ This platform has established protocols for evaluating gastrointestinal toxicity of pharmaceuticals, inflammation, wound repair, and fibrosis to assess therapeutic effects, drug delivery, and metabolism to predict drug permeation and absorption through the luminal side of the tissue.^[^ ^138^, ^141^ ^]^ In addition, host‐pathogen interactions, immune responses, and intestinal infections have also been investigated in these models to assess the effect of single species on intestinal cytoarchitecture and metabolism.^[^ ^138^, ^141^ ^]^ Although the model can mimic physiological conditions of the intestine, such as barrier integrity, metabolism, inflammation, and toxicity responses, it is necessary to consider the inoculation of intestinal microbiota to establish a host–microbiota research protocol over a more physiologically relevant window of time.^[^ ^138^, ^141^ ^]^

Treatment of patients with IBD (CD and UC) has continuously evolved in conjunction with advancing our understanding of disease etiology, and a personalized approach is now emphasized, shifting the goal of treatment from symptom relief to symptom remission via modulation of immune pathways related to disease etiology and pathogenesis.^[^ ^142^ ^]^ Before investigating precision medicine in vitro strategies, identifying hallmark disease progression biomarkers is essential for accurate diagnosis, distinguishment among classes of IBD, and predicting treatment efficacy.^[^ ^142f^ ^]^ In the case of intestinal microbiota conditions, there are many common biomarkers in clinical practice, such as serum markers (e.g., C‐reactive protein (CRP), glycoprotein (LRG)), fecal biomarkers (calprotectin and lactoferrin).^[^ ^142^, ^143^ ^]^ However, the translatability of these biomarkers is limited because they are not disease‐specific and broadly associated with various inflammatory disorders (e.g., CRP) or not FDA‐approved diagnostic tools for IBD (e.g., 16S), implying that clinical and in vitro diagnostics should consider exhaustive classification criteria that are comparable across studies.^[^ ^142^, ^143^, ^144^ ^]^ This also highlights the need for advanced bioinformatic tools to facilitate clinical application and refine diagnostic standards for IBD via microbiota signatures for improved translatability between in vitro and in vivo studies.^[^ ^143^, ^144^ ^]^

Currently, available treatments for IBD act on the host's immune modulations or preservation of healthy intestinal microbiota. Host modulation treatments (e.g., corticosteroids, aminosalicylates, immune modulators, and biologics), are administrated via oral or rectal modality; despite research advancements, these treatments are limiting because they focus solely on the level of inflammation and do not help initiate eubiosis amongst the intestinal microbiota.^[^ ^142^, ^144^ ^]^ Therefore, the need for combined therapies that target both the epithelial barrier and the microbiome would provide great benefits.^[^ ^145^ ^]^ Several innovative strategies for adjunct therapies focusing on initiating and maintaining microbiota eubiosis are currently under investigation in laboratory settings and clinical trials.^[^ ^142^, ^146^ ^]^ This includes personalized probiotic regimens and fecal microbiota transplantation (FMT), aimed at restoring balance to the microbiota rather than reducing inflammation.^[^ ^142^, ^147^ ^]^ Personalized probiotic regimes involve tailoring probiotic formulations to the specific imbalances observed within a patient's intestinal microbiota.^[^ ^148^ ^]^ FMT describes the transfer of stool from a healthy donor to an IBD patient to restore microbiota diversity.^[^ ^147^, ^148^, ^149^ ^]^ Current research focuses on optimizing donor selection and delivery methods and understanding the long‐term effects of the implementation into standard clinical practice.^[^ ^147^, ^148^, ^149^ ^]^ Only two fecal microbiota therapies have been approved by the FDA, including an enema and oral capsule; however, they are only used for the indication of recurrent *Clostridioides difficile* infection.^[^ ^149b^ ^]^

Future intestinal microbiota disorder research may focus on implementing diagnostic procedures that bridge the translational gap between clinical and in vitro results for increased outputs in drug discovery and development.^[^ ^142^, ^150^ ^]^ This includes advancing current in vitro models for the controlled investigation of therapeutics on various disease states.^[^ ^142c^ ^]^ Treatments should also continue to consider an adjunct approach to treating IBD. For example, focusing on both epithelial barrier health and restoring dysbiotic microbiome populations may further improve patient outcomes.^[^ ^142^, ^150^ ^]^

### 3.4 Female Reproductive Tract (FRT)

#### 3.4.1 Cyto‐Anatomical Architecture of the FRT Tissue: Host

**Figure 4:**
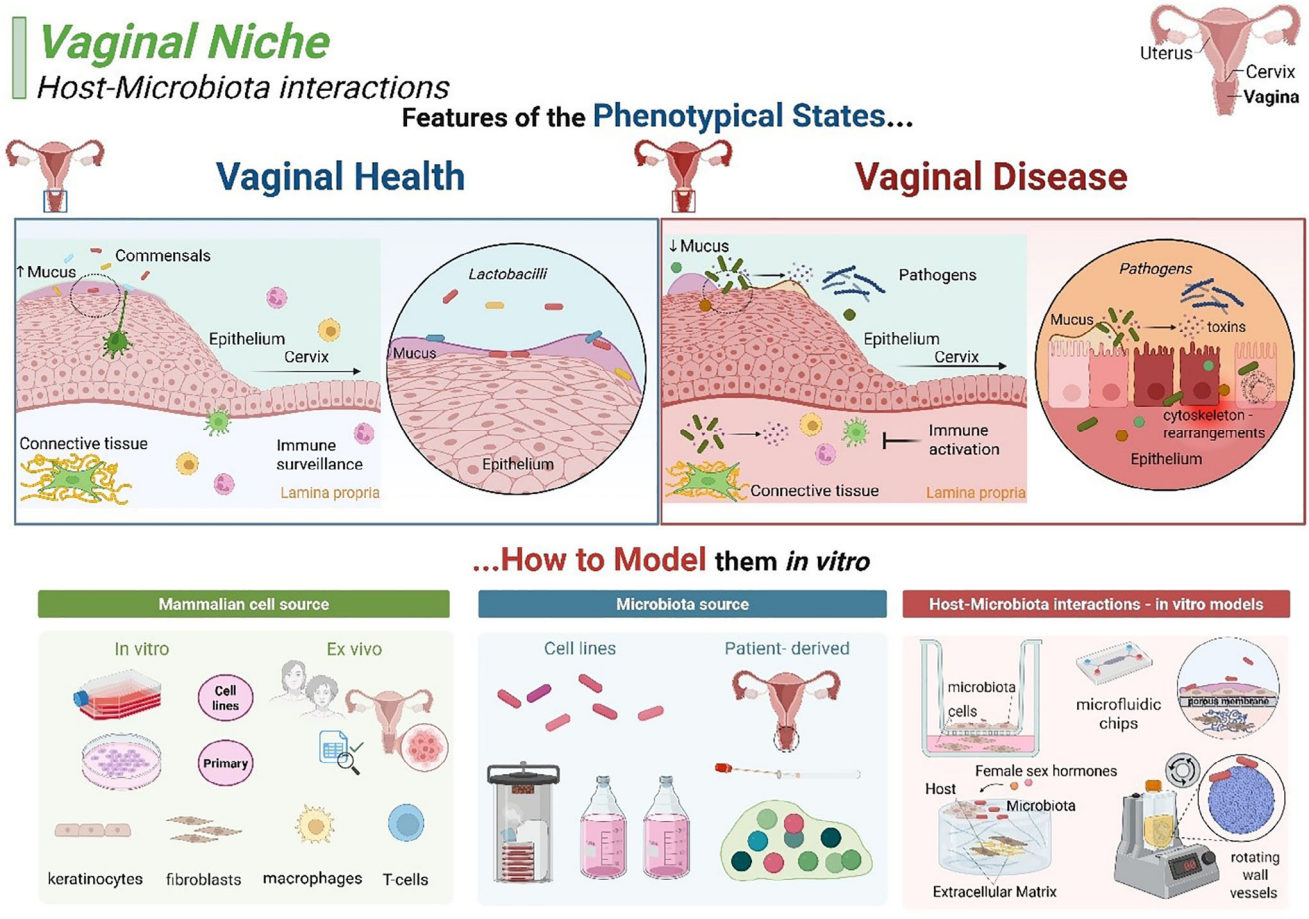
Vaginal niche. Upper – Representation of the vaginal niche in healthy and disease (Bacterial Vaginosis) states. Bottom – Overview of the mammalian and microbiota sources employed in current in vitro strategies. Created in BioRender. Ghezzi, C. (2025) https://BioRender.com/wq54d30.

The FRT (**Figure** **4**) includes several organ systems that are grouped in regions (upper and lower) and whose functions are involved in menstruation, fertility, procreation, and sexual activity during a woman's lifetime.^[^ ^151^ ^]^ Specifically, the upper region comprises endocervix, uterus, Fallopian tubes, and ovaries, while ectocervix and vagina refer to the lower region.^[^ ^3^, ^151^, ^152^ ^]^ In view of the level of detail of each tissue composing the FRT (cyto‐anatomical architecture and functions) required to describe each section, this review will focus only on the vaginal host and associated microbiota (vaginal microbiota), supported by the considerable literature available on vaginal in vitro models and vaginal microbiota. The vaginal tissue (Table 1) is composed of four layers: epithelium, lamina propria (sub‐epithelium), muscularis, and adventitia.^[^ ^3^, ^152^, ^153^ ^]^ The epithelium lining the vaginal tissue is a stratified squamous epithelium consisting of three sublayers (basale, suprabasal and apical cornified), the former characterized by proliferative cells that regenerate outer and senescent layers after shedding.^[^ ^152^, ^154^ ^]^ The suprabasal layer is rich in junctions (tight, adherence and desmosomes) and controlled permeability towards the lamina propria.^[^ ^155^ ^]^ The cornified apical layer is scarce in tight junctions and therefore highly permeable, facilitating the transport of high‐weight molecular mediators, such as immunoglobulins, and the interactions with commensal vaginal microbiota, important for preserving homeostasis.^[^ ^155^, ^156^ ^]^ Therefore, the native layered tissue architecture provides a barrier acting as a first line of defense against pathogen invasion in the lower region of the FRT.^[^ ^152^, ^153^, ^154^, ^157^ ^]^ The epithelium is supported by the underlying lamina propria, rich in connective tissue characterized primarily by collagen (type I, III and V) and elastic fibers embedded in a non‐fibrillar ECM (glycoproteins, hyaluronan and proteoglycans), and populated by vaginal fibroblasts and immune cells.^[^ ^153^, ^158^ ^]^ In connective tissue, collagens serve diverse functions; specifically, they provide tissue strength to support the overlying epithelium (type I), increase tissue flexibility and relaxation (type III), and lastly are involved in fibrillogenesis and wound healing (type V).^[^ ^158^ ^]^ Muscularis and adventitia follow the depth of the vaginal tissue, characterized by smooth muscle cells and loose connective tissue rich in large blood vessels, lymphatic system and innervation.^[^ ^153^ ^]^ To protect against harmful agents, the vaginal tissue has an immunological milieu (Table 2) residing in the lamina propria (monocyte/macrophages, Langerhans cells, dendritic cells, neutrophils, and B‐ and T‐lymphocytes) and in direct contact with the epithelium.^[^ ^152^, ^153^, ^156^, ^157^ ^]^ Different immunomodulatory defense mechanisms are in place in the vaginal tissue, such as immunoglobulins, antimicrobial peptides, proteases, and mucus layer.^[^ ^152^, ^156^ ^]^ Among these, the mucus layer protects the epithelium from external environmental factors and sexually transmitted agents, retains factors released by the host from underlying cells against pathogens, and houses of the vaginal microbiota.^[^ ^152^, ^153^, ^156^, ^157^, ^159^ ^]^ Mucus layer, generally referred as cervicovaginal mucus as it produced by the secretory vaginal epithelial cells and goblet cells at the cervix, is composed of structural proteins, mucins (MUC5AC, MUC5B), responsible for its viscoelastic properties, as well as immunoregulators and by‐products of the host and microbiota (reviewed in^[^ ^152^, ^156^, ^159^, ^160^ ^]^). Altogether, these mechanisms allow clearance of pathogens preventing possible vaginal infections and protecting other tissues from the lower region.

#### 3.4.2 Cyto‐Anatomical Architecture of the FRT Tissue: Microbiota

Compared to other niches in the body, the FRT microbiota exhibits the least richness and diversity and has a microbiota biomass of 10^11^ (microbes/person).^[^ ^3^, ^6^ ^]^ Vaginal microbiota is predominantly characterized by *Lactobacillus* species (anaerobic), such as *L. crispatus, L. gasseri, L. jensenii*, and *L. iners*.^[^ ^3^, ^152^, ^161^ ^]^ Although the relative abundance of *Lactobacillus* species might change within the female population, the vaginal microbiota is considered relatively stable.^[^ ^3^, ^152^, ^162^ ^]^ Differences may arise during different stages of women's life, especially during pregnancy, and are mainly driven by hormonal changes and menstruation.^[^ ^3^, ^162^, ^163^ ^]^ Vaginal microbiota has in fact been classified in five community state types (CST): CST (I) – dominated by *L. crispatus*; CST (II) – dominated by *L. gasseri*; CST (III) – dominated by *L. iners*; CST (IV A‐B) – dominated by non‐*Lactobacillus* dominant; CST (V) – dominated by *L. jensenii*. Generally, CST (I, II, V) are considered physiological vaginal microbiota, CST (III) presents higher vaginal inflammatory status and CST (IV A‐B) is considered not‐optimal vaginal microbiota with elevated inflammatory conditions, generally associated with bacterial vaginosis.^[^ ^3^, ^152^, ^159^, ^161^, ^163^, ^164^ ^]^ In the vagina, bacteria colonization happens at the epithelium level (Figure 4) and it is favored by mucus layer, low oxygen levels, and higher nutrient availability, highlighting the mutualistic interactions between host and its microbiota.^[^ ^159^, ^165^ ^]^ Bacterial adhesion is facilitated by glycan or mannose structures present in the mucus and by bacteria appendages (fimbriae) and mucin binding proteins.^[^ ^159^, ^160^, ^166^ ^]^ In addition to providing an ecological niche, the release of hormones (estrogen and progesterone) promotes the proliferation of *Lactobacilli* species at the mucus level, which in turn decrease vaginal pH, improve the vaginal epithelial barrier, and limit pathogen adhesion by occupying all available binding sites on vaginal epithelial cells.^[^ ^3^, ^159^, ^167^ ^]^

#### 3.4.3 FRT Host–Microbiota Interactions: Eubiosis and Dysbiosis

Species of *Lactobacillus* inhabit the vaginal niche and establish physiological interactions with vaginal epithelial cells promoting protective functions against foreign pathogens.^[^ ^3^, ^156^, ^157^, ^159^, ^167^, ^168^ ^]^ *Lactobacillus* species are the dominant bacteria found in the eubiotic vaginal microbiota, and their colonization, growth, functions, and interplay with the vaginal host are instructed by hormonal regulation during a woman's reproductive life and menstrual cycle (in order – proliferative, ovulatory, secretory and menstrual phases).^[^ ^3^, ^168^, ^169^ ^]^ Most of these mechanisms have been studied on *Lactobacillus crispatus* (CST‐I), whose relative abundance (as determined using 16S rRNA gene sequencing) corresponds to 26.2% of the total number of species analyzed in vaginal biopsy samples.^[^ ^3^, ^152^, ^162^, ^170^ ^]^ In the vaginal niche, the increase in abundance of *Lactobacillus* species begins at the end of the menstrual phase, when the surge in estrogen hormone (proliferative phase) favors their growth in the vaginal ecosystem.^[^ ^167^, ^169^, ^171^ ^]^ At this stage, estrogen acts directly on vaginal epithelial cells by enhancing their proliferation and maturation, as well as the production of L‐lactic acid and storage of glycogen in preparation for the secretory phase.^[^ ^3^, ^160^, ^164^, ^167^, ^169^ ^]^ Following ovulation, estrogen levels decrease, while progesterone levels rise, leading to epithelial lysis and release of previously stored glycogen.^[^ ^164^, ^165^, ^167^ ^]^ Glycogen secreted into the vaginal lumen is then catabolized by host and bacteria α‐amylase and α‐dextrins into smaller polymers (glycogen‐maltose‐maltotriose), which are subsequently fermented into L‐ and D‐lactic acid isoforms by *Lactobacillus* species.^[^ ^3^, ^167^ ^]^ *L. crispatus*, together with other *Lactobacillus* species, produces lactic acid, which has a central role in vaginal host–microbiota interactions.^[^ ^169^ ^]^ While lactic acid can be released by both epithelium (L‐isoform) and bacteria (L‐ and D‐isoforms), vaginal homeostasis is mainly driven by D‐lactic acid.^[^ ^172^ ^]^ D‐lactic acid promotes acidification of the vaginal microenvironment (pH<4), inhibiting the growth of pathogenic species, such as *Gardnerella*, *Prevotella* or *E. coli*, and it is suggested to reduce susceptibility to human immunodeficiency virus (HIV) infection.^[^ ^3^, ^159^, ^167^ ^]^ Furthermore, D‐lactic acid acts as a protective agent of vaginal tissue, as it improves the integrity of the vaginal epithelial barrier via the upregulation of tight junction proteins, and promotes mucus stabilization, important in shielding the epithelium from the external macroenvironment. In fact, *Lactobacillus* species lacks mucin‐degrading enzymes, favoring mucus stability and protecting the tissue from infections, in comparison to vaginal pathogens, such as *Gardnerella vaginalis*.^[^ ^160^, ^173^ ^]^ At the barrier level, given the ability of vaginal epithelial cells to produce L‐lactic acid, bacterial D‐lactic acid is protective of the cells from damage caused by their excessive lactic acid production through modulation of the extracellular matrix inducer metalloprotein (EMMPRIN‐MMP8); if not regulated, the activation of this pathway could lead to disruption to epithelial cells and subsequent entry of bacteria through the endocervix, leading to upper genital tract infections.^[^ ^172^ ^]^ Among the main functions of lactic acid, stabilization of the resident microbiota is one of the most important. Lactic acid has antimicrobial activity against non‐*Lactobacillus* species, viruses and protozoa, as it stimulates bacterial production of hydrogen peroxide (H_2_O_2_), and bacteriocins.^[^ ^159^, ^169^ ^]^ In particular, due to their cytoplasmic pH and osmotolerance, *Lactobacillus* species are not affected by lactic acid present in the vaginal environment, unlike other species, in which it causes acidification of their cytosol and increases their susceptibility to antimicrobial peptides or products released by the host and bacteria themselves.^[^ ^174^ ^]^ H_2_O_2_ can be used by the *Lactobacillus* species to compete with other species, especially catalase‐negative anaerobic organisms, to inhibit their growth.^[^ ^3^, ^159^, ^167^, ^175^ ^]^ Bacteriocins (Ia, IIc, J46, or type‐A lantibiotic), instead, play a role in controlling colonization and proliferation of dysbiotic‐associated bacteria and viruses, such as *Neisseria gonorrhoeae*, *Chlamydia trachomatis*, *Gardnerella vaginalis, Trichomonas vaginalis, Human Papillomavirus (HPV)* or *Herpes Simplex Virus (HSV)*.^[^ ^159^, ^164^, ^169^, ^176^ ^]^ In conjunction with the antimicrobial action driven by *Lactobacillus* species, the host cells release antimicrobial peptides (α/β defensins, elafin, cathelicidin), antibacterial enzymes (lactoferrin or lysozyme), and immunoglobulin (IgG), that can trap pathogen at the mucus layer, facilitating their clearance.^[^ ^168^, ^169^ ^]^ Ultimately, as a defense mechanism and stabilization of the healthy vaginal microbiota, lactic acid‐producing *Lactobacillus* species possess S‐layer binding proteins that facilitate their attachment to epithelial cells and thus compete with pathogens for adhesive receptors to prevent FRT infections.^[^ ^159^, ^177^ ^]^ While the microbiota functions rely on pH acidification, H_2_O_2_, and bacteriocins production, or competition for available binding sites on vaginal epithelial cells, host mechanisms comprise mucus layer structure and immunoregulation.^[^ ^3^, ^156^, ^157^, ^159^, ^167^, ^168^ ^]^ Increase in estrogen stimulates the production of cervicovaginal mucus that lines the vaginal epithelium, promoting colonization and stabilization of *Lactobacillus* species.^[^ ^167^ ^]^ The mucus layer has multiple functions: 1) it is a source of nutrient availability for the microbiota, 2) it forms a bridge between the microbiota and the epithelium, critical in establishing the vaginal epithelial barrier, protecting the entire FRT from foreign pathogens, and 3) it is a reservoir of immunoregulation by vaginal epithelial cells, vaginal immune cells, and the microbiota itself.^[^ ^152^, ^159^, ^160^, ^164^, ^169^ ^]^ During homeostasis and throughout the proliferative and secretory phases of the menstrual cycle, immunosuppressive mechanisms are in place to facilitate commensal vaginal colonization and preservation.^[^ ^167^, ^169^ ^]^ The vaginal immune system relies on TLRs, which are expressed on vaginal epithelial cells, but also on resident immune cells (macrophages, neutrophils, and dendritic and natural killer cells).^[^ ^156^, ^157^, ^168^ ^]^ Upon PAMPs recognition, TLRs promote secretion of cytokines as well as activation of neutrophils and leukocytes to remove dysbiotic bacteria.^[^ ^156^, ^157^ ^]^ To favor *Lactobacillus* species colonization, vaginal epithelial cells, and Langerhans cells suppress immune stimulation via release of Transforming growth factor‐β (TGF‐β) and cytokine secretion.^[^ ^156^, ^178^ ^]^ Increased anti‐inflammatory IL‐1RA and IL‐10 cytokines and decreased release of pro‐inflammatory of IL‐6, IL‐8, and TNF‐α cytokines have been reported in healthy host‐*Lactobacillus* interactions, indicating host modulatory mechanisms to favor vaginal colonization.^[^ ^3^, ^168^ ^]^ Specifically, release of TGF‐β, IL‐10, and IL‐15 inhibits T‐cells activation (helper and cytotoxic) and macrophages, promoting preservation of vaginal microbiota and suppressing target immune activation.^[^ ^156^ ^]^ At the end of the secretory phase, the separation of the endometrium from the uterine wall and subsequent shedding of the vaginal epithelium signify the beginning of the menstrual phase.^[^ ^164^, ^167^ ^]^ During this stage, the microbiota residing in the vaginal canal is highly diversified, similar to puberty stage (*Prevotella, Finegoldia, Peptoniphilus, Anaerococcus, Dialister*, and *Lactobacillus*) compared with the other phases of the menstrual cycle (proliferative, ovulatory, and secretory), with a decrease in *Lactobacillus* species and an increase in vaginal anerobic commensals.^[^ ^164^, ^179^ ^]^ This diversified microbiota is driven by a change in vaginal environmental conditions given by menstrual blood, which neutralizes acidic pH (7.2‐7.4), inhibits the antimicrobial activity of D‐lactic acid and increases availability of iron‐heme‐containing nutrient sources from damaged blood cells for iron‐dependent bacteria.^[^ ^180^ ^]^ These conditions favor vaginal anaerobic microbes, which are not able to thrive in acidic microenvironment driven by *Lactobacillus* species.^[^ ^179^ ^]^ Nevertheless, although hormonal regulation restores the vaginal acid environment at the end of the menstrual period, mediators of the host immune system, such as neutrophil gelatinase‐associated lipocalin (NGAL), prevent the subversion of the vaginal microbiota during menstruation by sequestering iron from iron‐dependent bacteria.^[^ ^164^, ^181^ ^]^ This mechanism allows re‐stabilization of proliferating *Lactobacillus* species upon restarting of the proliferative phase.^[^ ^182^ ^]^

In physiological conditions, vaginal health is preserved by the interplay of epithelium, microbiota, and immune cell surveillance.^[^ ^168b^ ^]^ However, impairment of the mucosal barrier, pro‐inflammatory responses and shift in microbial composition (depletion/overgrowth of *Lactobacillus* genera, associated with increase in relative abundance of anaerobic gram‐negative bacteria) can lead to clinical implications, such as bacterial vaginosis, cytolytic vaginosis, vulvovaginal candidiasis or pelvic inflammatory disease.^[^ ^3^, ^168^ ^]^ This review focuses on bacterial vaginosis (*Lactobacillus* genera depletion) because of its higher incidence and economic burden. However, it also aims to raise awareness of the persistence of supra‐optimal states due to overgrowth of commensal species, as in the case of cytolytic vaginosis (CV). CV is a vaginal infection with an incidence of 1.7‐16.3% that is often misdiagnosed as vulvovaginal candidiasis due to overlapping clinical signs and symptoms.^[^ ^3^, ^183^ ^]^ Currently, CV is defined by excessive epithelial lysis due to overproduction of lactic acid by *Lactobacillus spp*. resulting in a hyper‐acidic vaginal macroenvironment.^[^ ^184^ ^]^ Diagnostic criteria are based on pH, adhesion of *Lactobacillus spp*. to vaginal epithelial cells (false clue cells), lysed epithelial cells, absence of pathogenic bacteria, and vaginal discharge.^[^ ^185^ ^]^ Bacterial vaginosis (BV), instead, is a vaginal infection affecting 30% US women of reproductive age, causing, globally, a financial burden of 4.8 billion dollars.^[^ ^186^ ^]^ Symptoms range from vaginal discharge and burning sensation during urination, to swelling and itching, although in some case women appear to be asymptomatic.^[^ ^186^, ^187^ ^]^ Common phenotypical traits associated with BV comprise dysbiotic and polymicrobial communities (Table 3), change in vaginal pH, upregulation of pro‐inflammatory mediators with local inflammation and damage at the mucosa (epithelium and mucus).^[^ ^3^, ^169^, ^176^, ^188^ ^]^ The pathology displays a depletion in *Lactobacillus* species and an enrichment in anaerobic species (*Gardnerella*, *Atopobium*, *Megasphera*, *Prevotella*, *Porphyromonas*, *Peptostreptococcus*, and *Sneathia*), taxa that dominate in the CST IV group.^[^ ^152^, ^165^, ^169^, ^186^, ^187^, ^189^ ^]^ Among the dysbiotic bacteria, *Gardnerella vaginalis* showed high prevalence in women with the disease, but also in male partners, indicating possible sexual transmission.^[^ ^3^, ^190^ ^]^ At the onset of the disease, *G. vaginalis* acts as a colonizer of the vaginal epithelium, and, using fimbriae, firmly attaches to epithelial cells and lays down the biofilm.^[^ ^187^, ^189^, ^191^ ^]^ This step is necessary for the establishment of a polymicrobial biofilm (Table 4), as *G. vaginalis* serves as a bridge for the colonization of other anaerobic species, such as *Prevotella bivia* (early colonizer) or *Atopobium vaginae* and *Sneathia spp*. (secondary colonizers).^[^ ^187^, ^189^, ^192^ ^]^ In particular, *G. vaginalis* and *P. bivia* work synergistically on the vaginal epithelium in the early stages of the disease.^[^ ^193^ ^]^ Specifically, *Prevotella* species produce polyamines as part of their metabolism, which increases vaginal pH (>5) to promote the growth of other anaerobic species, as well as ammonia, which facilitates the growth of *G. vaginalis*. On the other hand, *G. vaginalis* synthesizes the necessary amino acids to promote the growth of *Prevotella*.^[^ ^187^, ^188^ ^]^ Upon colonization, *G. vaginalis* causes damage at the vaginal epithelial barrier.^[^ ^169^, ^188^ ^]^ Specifically, it releases the enzyme sialidase, which hydrolyzes sialic acid residues on the glycans of mucous layer lining the vaginal epithelium, leading to epithelial barrier breakdown.^[^ ^165^, ^194^ ^]^ In addition to barrier disruption, it releases pore‐forming toxic compounds, like vaginolysin, inducing cell lysis, that, in turn, will be used by bacteria as nutrient sources.^[^ ^188^, ^195^ ^]^ These mechanisms are also reinforced by polymicrobial communities, such as *Prevotella* species (*P. bivia* and *P. disiens*), which produce collagenase and fibrinolysins aiding mucosal breakdown, and *Atopobium vaginae* and *Sneathia* spp., which cause exfoliation of epithelial cells (clue cells).^[^ ^188^, ^196^ ^]^ Moreover, upon colonization, *G. vaginalis* can be internalized by vaginal epithelial cells and contributes to the remodeling of the cellular cytoskeleton. This uptake allows the bacteria to remain hidden from host immune surveillance, but also to regulate a pro‐inflammatory response on vaginal epithelial cells in exacerbating local inflammation and promoting the adhesion of other species.^[^ ^197^ ^]^ Increased levels of interleukin (1α/β, 6, and 12p70) and TNF‐α have been found in vaginal samples of women affected by BV.^[^ ^187^, ^198^ ^]^ In spite of the increase of pro‐inflammatory cytokines, enhanced release of bacterial enzymes, such as sialidase and prolidase, and bacterial metabolites belonging to short‐chain volatile fatty acids (SCFAs), like acetate or proprionate, leads to impairment of chemotaxis of immune cells, specifically neutrophils and monocytes.^[^ ^3^, ^199^ ^]^ Immunological markers of the disease, such as IL‐8, antimicrobial peptides (β‐defensin 1 and 2), and TLR, have been negatively correlated with increased levels of bacterial enzyme, as immune evasion mechanisms of dysbiotic bacteria, while enhanced levels of IL‐1β correlated positively with it.^[^ ^200^ ^]^ Taken together, these mechanisms incapacitate the host to clear pathogens while promoting colonization and growth of dysbiotic bacteria. Although our knowledge about the role of the microbiome in FRT has rapidly advanced, there is no clear understanding of the contribution of the microbiota in reproductive health and disease. Of the main problems is the relapse and recurrence seen in women treated with antibiotics for BV, which is one of the main focuses of vaginal research.^[^ ^3^, ^198^ ^]^ In absence of adequate animal models that can replicate human physiology and pathology in conjunction with microbiome composition, it is challenging to investigate the pathology and potential implications in pregnancy or preterm birth.^[^ ^3^, ^168^, ^198^ ^]^

#### 3.4.4 In Vitro Modeling of FRT Host–Microbiota Interactions

Several models (Figure 4) have been developed and validated to understand the contribution of the vaginal microbiota in reproductive health and disease (Table 5). Host models are based on primary vaginal epithelial cells and fibroblasts isolated from vaginal biopsies or immortalized cell lines; moreover, fibroblast cells from uterine biopsies or skin fibroblast cell line have been used as alternative sources.^[^ ^10^, ^22^, ^173^, ^201^ ^]^ To display characteristics of mature vaginal keratinocytes, previously characterized immortalized cell lines derived from normal epithelial tissue via hysterectomy (VK2/E6E7) have been extensively employed in vaginal research, as they express phenotypical and functional traits of vaginal epithelium (cytokeratin markers, multilayered structure, bacterial binding characteristics, TLRs and hormonal responses).^[^ ^201^, ^202^ ^]^ In vitro recapitulation of the vaginal features has been achieved by culturing vaginal epithelial cells on tissue culture plates or Transwell supports.^[^ ^173^, ^201^ ^]^ Transwell systems were also employed to study functional responses of vaginal epithelial cells grown on laminin coating in conjunction with peripheral blood mononuclear cells.^[^ ^203^ ^]^ Lastly, the integration of fibroblasts grown on top of the collagen matrix in vaginal epithelial cultures or the enhancement of three‐dimensional structures by microspheres or Whatman paper to form collagen stromal sheets helped to achieve the complexity of cytoarchitecture shown in vivo.^[^ ^10^, ^22^, ^204^ ^]^ Overall, these models have successfully replicated the stratification and differentiation of vaginal epithelium (Cytokeratins, Involucrin), barrier formation through the expression of junctional complexes (E‐Cadherin, Zonula Occludens‐1 or Desmoglein), and mucus formation (Mucins). Moreover, as menstrual hormones and lactic acid functionally regulate vaginal epithelial architecture and response as well as microbiota colonization, current strategies have been proven responsive to stimulation with female sex hormones (estradiol or progesterone) or lactic acid.^[^ ^173^, ^201^, ^205^ ^]^ In vivo vaginal features (microfolds, microvilli, mucus formation, barrier integrity permeability) and incorporation of immune cells (T‐cells and macrophages) have improved through the integration of flow stimulation employing rotating wall vessels (RWV) via encapsulation of cells into microcarrier beads or organ‐on‐a‐chip technologies, which used coating of collagens and poly‐L‐lysine.^[^ ^10^, ^206^ ^]^ Transmission electron microscopy of immortalized vaginal cells cultured in RWV indicated secretory vesicles/mucus deposits, suggesting the ability of these cells to be involved in mucus secretion. In addition, linked cervical and vaginal organ‐on‐a‐chip have proven useful in mimicking cervico‐vaginal mucus lining the vaginal mucosa through shear simulation of primary human cervical epithelial cells.^[^ ^10^, ^207^ ^]^

As the vagina is also the FRT's first line of defense,^[^ ^152^ ^]^ host–microbiota interactions have been focused on reproducing vaginal health by utilizing patient‐derived microbiota or selected bacterial cell lines to study epithelial colonization, tissue response and phenotypic changes to pathogens causing infection or sexually transmitted diseases (STDs).^[^ ^10^, ^201^, ^204^, ^208^ ^]^ Lastly, to investigate tissue regeneration after wound healing and accelerate the testing and commercialization of targeted drugs or therapies aimed at restoring physiological host–microbiota balances (depletion/overgrowth of physiological microbiota), 2D, 3D, or commercially available models (e.g., EpiVaginal™) have also been developed.^[^ ^10^, ^201^, ^205^, ^209^ ^]^ Collectively, host–microbiota readouts are centered on tissue architecture (stratification, differentiation, barrier properties, mucus and microvilli),^[^ ^10^, ^201^, ^203^, ^204^ ^]^ microbial colonization or invasion and,^[^ ^10^, ^201^, ^204^ ^]^ pro‐ and anti‐inflammatory response,^[^ ^10^, ^201^, ^203^, ^204^ ^]^ pH and lactate levels,^[^ ^10^, ^173^ ^]^ female sex hormones stimulation and responses,^[^ ^201^, ^205^ ^]^ and drug testing.^[^ ^10a^ ^]^

#### 3.4.5 Emerging Clinical Strategies Targeting FRT Host–Microbiota Interactions in Disease States

Vaginal microbiota‐related disorders, including BV and pelvic inflammatory disease (PID), have available treatments that are limited primarily to antibiotics.^[^ ^210^ ^]^ BV is generally treated with prescribed oral and intravaginal antibiotics, such as Metronidazole and Clindamycin. At the same time, the CDC recommends Doxycycline and Cephalosporin (e.g., ceftriaxone, cefoxitin, or probenecid) for PID that target broad‐spectrum bacteria (Gram‐positive and Gram‐negative).^[^ ^210b^ ^]^ In the case of failed outpatient treatment for PID, intravenous antibiotics are recommended.^[^ ^210b^ ^]^ Antibiotic treatments, however, can increase the chance of recurrence of BV due to a failure of *Lactobacillus* species to recolonize the vagina or cause antibiotic resistance, as shown for *G. vaginalis*, a pathogenic species involved in BV.^[^ ^189^, ^211^ ^]^ A late diagnosis due to the lack of symptoms is a current main challenge for precision medicine advancements, particularly for PID.^[^ ^210b^ ^]^ This emphasizes the need for improved diagnostics via biomarker discovery and standardization of screening for antibiotic resistance to determine the appropriate antibiotics to prescribe for pathogen‐specific targeting.^[^ ^189^, ^211^ ^]^ Consequently, to advance therapeutic development targeted at vaginal‐microbiota‐related disorders, it is essential to develop physiologically relevant in vitro models for accurate disease modeling of various vaginal conditions and improved clinical relevance.^[^ ^212^ ^]^ Commercially available models, such as the EpiVaginal^TM^, have proven helpful in assessing some of the mechanisms of inflammation in vitro, such as in the case of the mode of action of vaginolysin (VLY), a toxin produced by *G. vaginalis*, which can mediate BV‐damage via infiltration of basolateral and not‐apical epithelium.^[^ ^212^, ^213^ ^]^ Despite these key findings, commercially available models require further improvement to be considered physiologically relevant. In particular, the absence of immune components and lack of dynamic stimuli do not facilitate physiological colonization, biofilm formation, and growth of healthy vaginal microbiota species but instead promote a basic pH (7.2), deviating from accurate clinical findings.^[^ ^213^, ^214^ ^]^ An additional shortcoming in the design of physiological in vitro models is the accurate modeling of vaginal disease progression across the lower and the upper regions of the FRT, as for PID. Progression of PID includes infection, inflammation, tissue damage, and scarring stages, and the disease can spread from the vagina to the Fallopian tubes, posing the challenge of modeling multiple tissues in a single in vitro platform.^[^ ^210^, ^212^, ^213^ ^]^ Developing in vitro models for PID is complex. Still, micro‐physiological systems offer promises by enabling co‐culture of various tissue types (e.g., uterus, fallopian tubes, ovaries) and replicating physiological interactions.^[^ ^215^ ^]^ To address the need for alternative therapeutics, several precision medicine approaches are being investigated in vitro and in vivo for the treatment of BV, such as probiotics, vaginal microbiota transplantation (VMT), pH modulation (boric acid or lactic acid), biofilm disruptors, and modification of lifestyle factors.^[^ ^211^, ^216^ ^]^ The application of probiotics vaginally is being investigated for the recolonization of healthy vaginal microbiota.^[^ ^211^, ^216^ ^]^ Similar output has been achieved with the VMT, which supports the healthy microbiota recolonization through the transferal of the whole vaginal microbiota of a healthy donor to a patient with dysbiotic vaginal microbiota^[^ ^211^, ^216^ ^]^ On the other hand, pH modulators, such as lactic acid supplementation, are central in counteracting the decrease in lactic acid production observed in BV patients, as they work to restore the acidic and healthy pH driven by the microbiota (pH <5).^[^ ^211^, ^216^ ^]^ In the case of recurrent BV, which is believed to be due to enhanced dysbiotic microbial biofilms, alternative approaches are being investigated to disrupt these biofilms.^[^ ^211^, ^216^ ^]^ For example, genetically engineered endolysins targeted to destroy *Gardnerella* biofilms have succeeded in vitro pre‐clinical studies, but have not yet been investigated in vivo.^[^ ^211^, ^216^ ^]^ Implementing these therapeutics to treat and prevent the recurrence of BV may also reduce the occurrence of PID, as often indicated as comorbidities.^[^ ^211^, ^216^, ^217^ ^]^

Enhancing combined treatments such as prebiotic and probiotic regimes or VMT to support the recolonization of healthy microbiota shows promise in mitigating the recurrence of BV.^[^ ^211^, ^216^ ^]^ The standardization of in vitro models for correlative studies and reproducibility will address the need for drug development targeted at vaginal microbiota‐related disorders.^[^ ^212^, ^213^, ^214^, ^215^, ^216^, ^218^ ^]^ With this, developing a deeper understanding of disease pathogenesis can lead to revolutionary personalized or precision medicine approaches tailored to an individual's unique microbiome, hormone levels, and lifestyle factors to improve the treatment success rate.^[^ ^211^, ^217^ ^]^

## 4 Conclusive Remarks and Future Perspectives

Our current understanding of microbiota research has rapidly progressed in recent decades. Clinical (human and animal) and in vitro investigations have successfully advanced research on the distinct topographic interfaces that allow microbes to form stable ecosystems in which the community can thrive, interact, and evolve in a mutualistic manner with the host.^[^ ^1^, ^2^ ^]^ Systemic communication between the host and its microbiota regulates tissue physiology, metabolism, immune response, barrier integrity on mucosal surfaces, as well as microbial composition, networking, and pathogen surveillance.^[^ ^1^, ^2^, ^3^, ^27^, ^110^, ^152^ ^]^ However, perturbations of this finely regulated crosstalk in the ecological niche, as well as age, stress level, immune impairment, drug use, smoking, hygiene or dietary habits,^[^ ^235^ ^]^ can cause a loss of commensals colonization, the thriving of pathogenic bacteria, tissue inflammation, and damage to mucosal surfaces, which collectively are enhanced drivers of disease.^[^ ^1^, ^2^, ^3^, ^27^, ^28^, ^29^, ^85^, ^111^, ^152^, ^168^ ^]^ Yet, the interactions among different players (host, immune cells, and microbiota) along with environmental factors that may explain disease onset or relapse/ recurrence have not been clarified. Among the above‐mentioned factors, emerging evidence is accounting for the impact of sex, race, and ethnicity on microbiota composition and, thereby, relative interactions with the host. It has been reported that the female skin microbiota is characterized by a higher species diversity than that of males,^[^ ^236^ ^]^ while the oral niche is more sensitive to hormonal fluctuations, which affect immune responses on mucosal surfaces and alter saliva composition. This is associated with increased risk factors for the female sex and secondarily with age due to hormonal fluctuations through the menstrual cycle, pregnancy, and menopause.^[^ ^237^ ^]^ The intestine niche instead harbors estroblome, a collection of bacteria that can metabolize estrogens by deconjugating estrogen compounds via the enzyme β‐glucuronidase, which can then recirculate and impact hormonal balance in the human body. This contributes to sex‐based differences in the intestinal microbiota in conjunction with environmental, and sociocultural factors associated with age, race, and ethnicity contributing to variations in metabolic processes and therefore to microbiome composition.^[^ ^238^ ^]^ Lastly, hormonal fluctuations also influence the FRT niche. Estrogen is known to play a crucial role in maintaining a healthy, Lactobacillus‐dominant vaginal microbiota, and it has been shown that hormonal contraception can alter Lactobacillus dominance. Other factors such as race and ethnicity have been found to have an impact on the vaginal microbiota composition, marked by women of African or Hispanic descent having relatively lower counts of Lactobacillus species and increased counts of Gardnerella species in comparison to White and Asian women.^[^ ^239^ ^]^ These considerations on the impact of sex, race, and ethnicity in influencing host‐microbiome signatures, underscore the need to incorporate these variables into experimental designs for more accurate and representative studies. Most engineered models reported in this review are designed to represent simplified environments, replicating anatomical or microbial systems without incorporating sex‐specific or population‐specific variations.^[^ ^10^, ^16^, ^25^, ^214^ ^]^ Future experimental designs should consider these biological and sociocultural variables by stratifying donor samples (e.g., microbiota, tissue, or cell lines) based on sex, race, and ethnicity, adopting hormone supplementation to mimic sex‐specific environments, and integrating genetic or immune variations that model subsets of populations.

**Figure 5:**
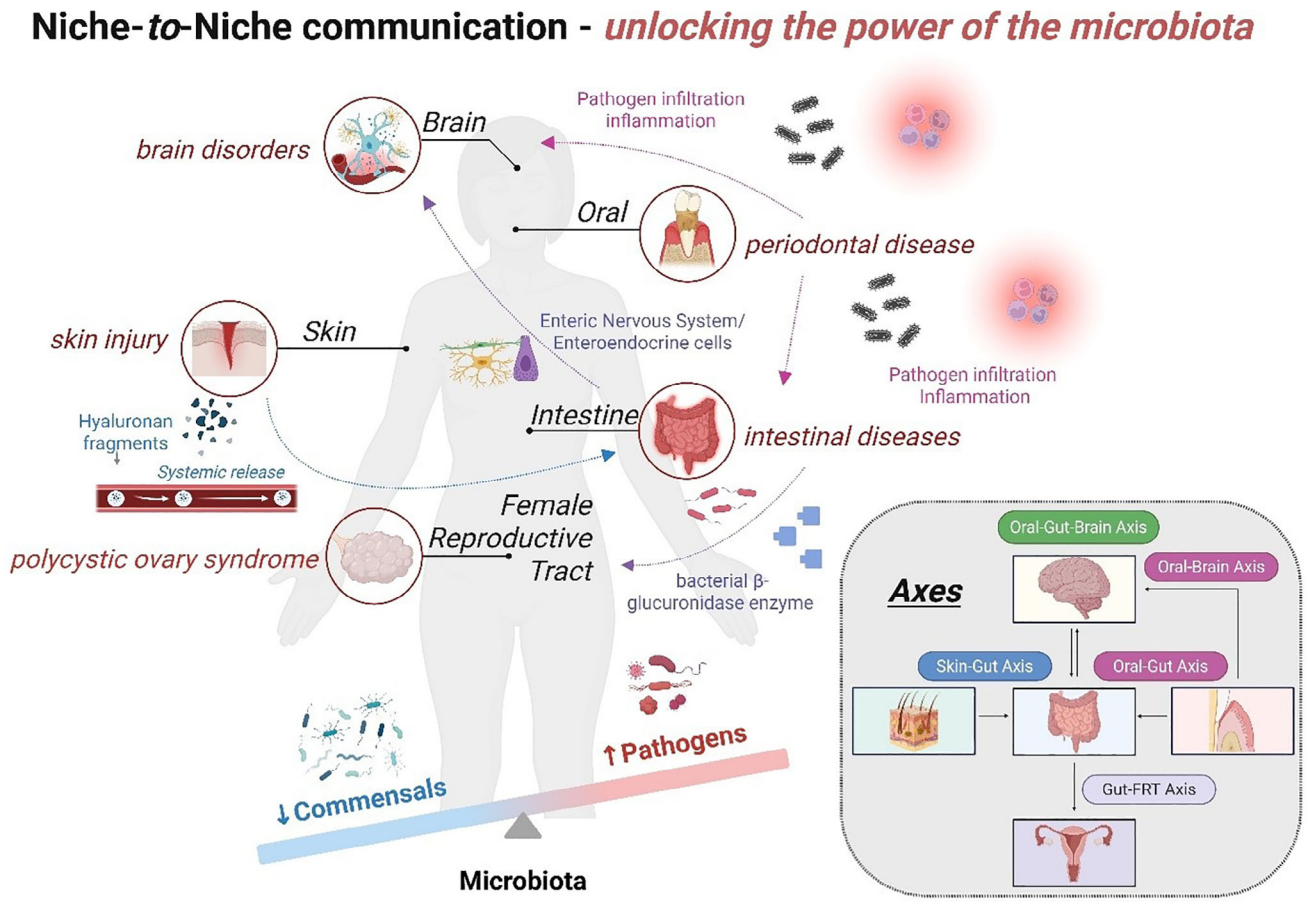
Niche‐to‐Niche communication. Systemic role of the dysbiotic microbiota in the human body. Created in BioRender. Ghezzi, C. (2025) https://BioRender.com/mw3jkfs.

Beyond the concept of beneficial interaction between the host and its microbiota, studies on microbial niches are uncovering the influence of the microbiota at distal sites through the release of bacterial secondary metabolites or translocation of bacteria/pathogens into the bloodstream, supporting a systemic role of the microbiota in the human organism (**Figure** **5**).^[^ ^240^ ^]^ Various examples of systemic effects throughout the body stem from the intestinal niche. Synthesis of SCFAs (acetate, butyrate, lactate, or propionate), known to physiologically regulate intestinal homeostasis and prevent colonization of harmful bacteria through immune and host regulation, can be used as energy sources by the liver, influence the growth and differentiation of neurons in the brain and distally influence skin commensal *S. epidermidis* and *C. acnes* colonization.^[^ ^241^ ^]^ Furthermore, intestinal microbiota communicates with skin tissue by participating in skin allostasis after UV treatment, enhancing T‐cell differentiation processes, and also with vaginal tissue through the metabolization and circulation of the active form of estrogen hormone by the bacterial enzyme β‐glucuronidase.^[^ ^241^, ^242^ ^]^ Although verified mechanisms of beneficial contribution to human health by the microbiota have been reported, the clinical significance of these niche dialogues is emerging in pathological conditions, where tissue perturbations in the commensal/pathogen ratio can lead to systemic inflammation and dysbiosis at distal sites.^[^ ^1^, ^2^, ^3^, ^27^ ^]^ Clinical indications have been reported for the following axes: skin‐intestine, intestine‐vagina, and oral‐intestine‐brain. Systemic release of hyaluronan fragments during skin injury induces intestinal fibroblasts differentiation to pro‐inflammatory adipocytes, which, in turn, cause intestinal inflammation leading to dysbiosis (skin‐intestine axis).^[^ ^243^ ^]^ Reduction of bacterial β‐glucuronidase enzyme in the intestine might be associated with pathogenic states, such as obesity or polycystic ovary syndrome (intestine‐FRT axis).^[^ ^244^ ^]^ Dysbiotic oral microbiota (*Fusobacterium, Porphyromonas, Veillonella, Treponema* and *Campylobacter*) can enter the bloodstream via tissue lesions leading to: 1) intestinal dysbiosis – IBD or colorectal cancer – (oral‐intestine axis) due to bacterial protease release (e.g., gingipain), disruption of intestinal epithelial barrier, and immune dysregulation; 2) brain disorders – cognitive disorders or neurodegenerative diseases (e.g., Alzheimer's disease) – (oral‐brain axis) through colonization by *P. gingivalis* of coronary and femoral arteries, resulting in acute systemic and brain inflammation via increased permeability and disruption of the blood‐brain barrier and immune activation (microglia); 3) intestinal and brain disorders (oral‐intestine‐brain axis) via enteroendocrine (enterochromaffin) cells and mesenteric and vagus nerve afferent fibers in the intestine.^[^ ^85^, ^106^, ^245^ ^]^ Although most of these clinical considerations are derived from observational data in clinical or animal studies, advances in tissue engineering are currently emerging, supporting longitudinal analysis of these bidirectional pathways. Intestinal microbe‐derived metabolites have been shown to improve neural differentiation and maturation (synaptogenesis and synaptic plasticity) in human‐induced neuronal stem cells cultured in an intestine‐brain axis chip.^[^ ^246^ ^]^ Furthermore, inoculation of intestinal‐derived SCFAs (butyrate and valerate) on inflamed monoculture of glioblastoma astrocytoma and neuroblastoma cell has been shown to modulate neuroinflammatory and neurodegenerative processes.^[^ ^247^ ^]^ Impairment of skin health as a result of increased intestinal barrier permeability due to absorption of free fatty acids (palmitic acid) has been demonstrated in the intestine‐skin axis chip with a substantial decrease in viability and increased inflammatory profile of skin cells.^[^ ^248^ ^]^ Indications of the colonization of oral species in the intestine by mucin proteins have been demonstrated in the Mucosal ARtificial COLon bioreactor (M‐ARCOL), although in absence of host cells, in which the colonization of salivary microbiota on mucosal surfaces versus luminal surfaces suggested potential geographic competition between oral and intestinal microbial population.^[^ ^249^ ^]^ In this light, given the growing evidence of niche‐to‐niche communication, mechanisms related to the clinical potential and translatability of therapeutic agents between axes are being investigated. This emphasizes the importance of viewing the body's microbiota niches as an integrated system rather than a collection of isolated parts when considering the development of therapeutic approaches.^[^ ^217a^ ^]^ Taking advantage of these bidirectional communications, current investigations utilize precision medicine strategies to further develop multi‐target host–microbiota modulation therapies (e.g., probiotic, prebiotic, postbiotic supplementation, and microbiota transplants).^[^ ^250^ ^]^ For example, supplementation of healthy bacteria, such as probiotics,^[^ ^100^, ^251^ ^]^ has been shown to reduce inflammation and restore microbial balance for symbiotic communication between niches, as in oral‐intestine and intestine‐vaginal niches.^[^ ^217^, ^250^, ^252^ ^]^ Oral administration of probiotics as a treatment for oral and intestinal microbiota disorders has shown in clinical trials a similar beneficial effect on vaginal microbiota health compared to direct vaginal administration.^[^ ^253^ ^]^ The mechanism has been attributed to the restoration of the health of the intestinal microbiota, which positively impacts the vaginal microbiota (intestine‐vagina axis), causing the attenuation of urogenital pathogens (*E. coli*) in the intestine due to the acidic environment created by lactic acid‐producing probiotics (*L. rhamnosus* and *L. reuteri*).^[^ ^253^ ^]^ A similar positive systemic effect was demonstrated on the intestine‐brain axis, where probiotics administered orally for indications related to the intestinal microbiota improved cognitive functions.^[^ ^4^, ^254^ ^]^ This is attributed to the enhanced epithelial barrier and blood–brain barrier (BBB) function via lowered systemic lipopolysaccharide (LPS) levels, mitigating inflammation and thought to reduce glial activation, ultimately lowering the risk of neurodegenerative disease.^[^ ^4^, ^254^ ^]^ Like probiotics, FMTs represent another leading strategy in precision medicine treatments, as they reduce neuroinflammation and improve cognitive function and ability in patients with Alzheimer's disease, Parkinson's disease, multiple sclerosis, and amyotrophic lateral sclerosis, although there is no consensus on the results yet.^[^ ^4^, ^254^ ^]^ Leveraging the bidirectionality of these communications is critical to revolutionize therapeutic development, as it enables treatments targeting one microbial niche to have systemic effects.^[^ ^217^, ^253^ ^]^ Therefore, it is imperative to decipher the mechanisms of interaction between the host and its microbiota, as well as how the ecological balance within the niche adapts to the host's lifestyle or changes during disease onset. This new perspective should be the foundation of future research approaches to develop effective treatments and prevention strategies for the treatment and prevention of complex human diseases such as systemic inflammation, infection, or neurodegenerative disorders.^[^ ^217^, ^253^ ^]^ Successful strategies have analyzed microbial composition using 16S rDNA sequencing or microbial biogeography by fluorescence in situ hybridization (FISH) to categorize bacteria as healthy‐ or disease‐associated or to decode communication and assemblage within the biofilm.^[^ ^3^, ^16^, ^27^, ^81^ ^]^ In addition, clinical investigations are developing standardized methodologies to capture disease onset by elucidating key mechanisms related to social interactions, inflammatory profiles, and commensal/pathogen abundance and ratio.^[^ ^3^, ^71^ ^]^ However, many questions remain unanswered regarding the biological mechanisms and temporal profile that drive dysbiosis or disease relapse.

Although knowledge of microbial composition in every niche of the human body has rapidly advanced through 16S rDNA analysis and metagenomics, functional information on microbial consortia is still very limited. In this light, in vitro models offer advantageous opportunities to study the ecology and mechanisms that regulate host–microbiota interactions in each ecological niche, as they allow the identification of early biological mechanisms or biomarker profiles that could precede disease initiation. Current in vitro strategies are based on recapitulation of some of the major physiological features of the tissue of interest; however, there are still some shortcomings that limit the long‐term functionality of in vitro models: (1) failure to integrate full‐thickness tissue architecture and absence of resident immune, vascular or nerve cells in biomaterial‐based models; (2) use of planktonic bacteria or selected species, leading to misrepresentation of microbial communities, communication and translatability of results; (3) failure to reproduce mechanical and physical properties and/or selective forces that model the ecological niche; and (4) inadequate culture conditions, such as saliva (oral), dryness (skin) or moisture (FRT). To advance the development of physiologically relevant in vitro models and support personalized diagnosis and treatments, advances in computational or mathematical modeling and bioinformatics techniques are currently being pursued to address current challenges in large data sets. Machine learning and artificial intelligence are exhibiting great potential in tissue engineering strategies; through the generation of algorithms (e.g., deep learning), existing data can be used to train the model, thereby informing experimental parameters, such as material design and properties, material biocompatibility, or cell classification, which are ultimately used to mimic host responses and microbial population behaviors with high fidelity.^[^ ^255^ ^]^ Mathematical models (e.g., game theories) are used to model host‐pathogen interactions to understand whether a pathogen is aggressive or non‐aggressive toward a specific cell type.^[^ ^256^ ^]^ Ultimately, the integration of computational biology and machine learning in respect to multi‐omics data (e.g., meta‐genomics, meta‐transcriptomics, meta‐proteomics, and metabolomics) can serve as a tool to analyze and integrate heterogeneous data with the ultimate goal of 1) understanding host–microbiota interactions; 2) generating and testing new hypotheses; and 3) obtaining phenotypic prediction of biomarkers for early detection of host–microbiota imbalances for clinical applications.^[^ ^257^ ^]^

Multidisciplinary efforts will be paramount to investigate health and disease states from different perspectives. Physiologically relevant tissue models are key to recreate the host–microbiota balance, if successfully designed. By incorporating tissue cyto‐anatomical architecture, patient‐derived microbiota, along with tissue mechanics, physics, and metabolic conditions, tissue models can provide agile tools where engineering design meets omics and high‐resolution imaging of living cells; these high‐throughput systems can enable high‐precision unraveling of tissue homeostasis, disease states, and treating, or even anticipating, disease‐associated outcomes. Moreover, given niche‐to‐niche communication, multi‐organ tissue models will be paramount to unlock the power of the microbiota in regulating human health.

## Conflict of Interest

The authors declare no conflict of interest.

## Author Contributions

M.A. and C.E.G. conceived and designed the manuscript. M.A., G.E.C., and C.E.G. wrote the manuscript. M.A. and C.E.G. conceptualized and designed the cartoons. M.A., G.E.C., and C.E.G. performed editing and review. X.H., B.J.P., and H.H. performed formal editing and provided feedback. C.E.G. provided funding acquisition.
